# Lysosomal TRPML1 regulates mitochondrial function in hepatocellular carcinoma cells

**DOI:** 10.1242/jcs.259455

**Published:** 2022-03-21

**Authors:** Wei Xiong Siow, Yaschar Kabiri, Rachel Tang, Yu-Kai Chao, Eva Plesch, Carola Eberhagen, Florian Flenkenthaler, Thomas Fröhlich, Franz Bracher, Christian Grimm, Martin Biel, Hans Zischka, Angelika M. Vollmar, Karin Bartel

**Affiliations:** 1 Department of Pharmacy, Center for Drug Research, Pharmaceutical Biology, Ludwig-Maximilians-University of Munich, 81377 Munich, Germany; 2 Technical University Munich, School of Medicine, Institute of Toxicology and Environmental Hygiene, Biedersteiner Strasse 29, 80802 Munich, Germany; 3 Walther-Straub-Institute of Pharmacology and Toxicology, Ludwig-Maximilians-University Munich, 80336 Munich, Germany; 4 Department of Pharmacy, Center for Drug Research, Pharmaceutical Chemistry, Ludwig-Maximilians-University Munich, 81377 Munich, Germany; 5 Institute of Molecular Toxicology and Pharmacology, Helmholtz Center Munich, German Research Center for Environmental Health, Ingolstaedter Landstrasse 1, 85764 Neuherberg, Germany; 6 Gene Center, Laboratory for Functional Genome Analysis, Ludwig Maximilians-University Munich, 81377 Munich, Germany; 7 Department of Pharmacy, Center for Drug Research, Pharmacology, Ludwig-Maximilians-University Munich, 81377 Munich, Germany

**Keywords:** TRPML1, Hepatocellular carcinoma, Mitochondria, Lysosome, Cancer, Ca^2+^, Mitophagy

## Abstract

Liver cancers, including hepatocellular carcinoma (HCC), are the second leading cause of cancer death worldwide, and novel therapeutic strategies are still highly needed. Recently, the endolysosomal cation channel TRPML1 (also known as MCOLN1) has gained focus in cancer research because it represents an interesting novel target. We utilized the recently developed isoform-selective TRPML1 activator ML1-SA1 and the CRISPR/Cas9 system to generate tools for overactivation and loss-of-function studies on TRPML1 in HCC. After verification of our tools, we investigated the role of TRPML1 in HCC by studying proliferation, apoptosis and proteomic alterations. Furthermore, we analyzed mitochondrial function in detail by performing confocal and transmission electron microscopy combined with Seahorse^TM^ and Oroboros^®^ functional analysis. We report that TRPML1 overactivation mediated by a novel, isoform-selective small-molecule activator induces apoptosis by impairing mitochondrial function in a Ca^2+^-dependent manner. Additionally, TRPML1 loss-of-function deregulates mitochondrial renewal, which leads to proliferation impairment. Thus, our study reveals a novel role for TRPML1 as regulator of mitochondrial function and its modulators as promising molecules for novel therapeutic options in HCC therapy.

## Abstract (precis)

**Summary:** The endolysosomal ion-channel TRPML1 is a regulator of mitochondrial function; an isoform-selective activator modulates TRPML1 channel activity and potentially could treat liver cancer.

## INTRODUCTION

Liver cancers are the fourth most common and second leading cause of cancer death worldwide, with a still rising incidence. The World Health Organization estimates that there will be over one million annual deaths caused by liver cancer by the year 2030 (Villanueva, 2019). Hepatocellular carcinoma (HCC) is the most common type of primary liver cancer and hence is a global health problem (Yang et al., 2019). Despite the severity of the disease and intensive research, pharmacologic therapies for HCC are still limited. To date, only kinase inhibitors like sorafenib or lenvatinib are clinically used for HCC therapy (Kudo et al., 2018; Llovet and Bruix, 2008). Even though clinical management of HCC has improved in the last years and several trials testing immune-based therapies show promising results so far, there is still a high need to develop novel therapeutic strategies to fight HCC (Villanueva, 2019).

In the past decade, lysosomes have gained interest in cancer research. Since their discovery as cellular recycling centers, lysosomes have emerged as complex cellular signaling hubs, participating in the regulation of cell growth, division, differentiation and cell death in a physiological, as well as a pathophysiological context (Lawrence and Zoncu, 2019; Wang et al., 2018). Therefore, lysosomes are now considered as interesting targets in treating many different diseases. Especially, in terms of cancer treatment lysosomes have come into focus in recent research (Davidson and Vander Heiden, 2017; Piao and Amaravadi, 2016; Saftig and Klumperman, 2009). Lysosomal membrane proteins, namely ion channels and transporters, critically regulate lysosomal functions, hence they are promising candidates as novel drug targets. The acidic lysosomal lumen is provided by the vacuolar H^+^-ATPase (V-ATPase), which is by now an established anti-cancer target (Bartel et al., 2017; Cotter et al., 2015; Forgac, 2007; Kubisch et al., 2014; Schempp et al., 2014; von Schwarzenberg et al., 2013, 2014). Besides the V-ATPase, different families of cation channels can be found in the lysosomal membrane. Among them, the TRPML (mucolipins) and the two-pore channels (TPCs) have been reported to play essential roles in exocytosis processes, endolysosomal trafficking and autophagy. Recently, these channels have also been reported to be important for various diseases, yet data on the role of TRPML1 (also known as MCOLN1) in cancer is just emerging (Chao et al., 2017; Grimm et al., 2014; Kiselyov et al., 2012; Müller et al., 2021; Nguyen et al., 2017; Sakurai et al., 2015; Santoni et al., 2020; Shen et al., 2012). The mucolipins consist of TRPML1, TRPML2 (MCOLN2) and TRPML3 (MCOLN3), which are cation channels belonging to the TRP channel family. While TRPML2 and TRPML3 are expressed in specialized cells (e.g. immune cells or melanocytes), TRPML1 is broadly expressed and mainly localized in the lysosomes, where it promotes cation efflux and hence is implicated in lysosomal storage, transportation and pH homeostasis (Morgan et al., 2011). In cancer, TRPML1 overexpression results in faster growth and improved survival of cells for several cancers, whereas activation of the channel has been reported to be beneficial, for instance, in glioblastoma (Morelli et al., 2019; Santoni et al., 2020). Mechanistically, TRPML1 function has been shown to be crucial for autophagy regulation. On the one hand, activation of TRPML1 has been reported to disrupt the autophagic process and induce cell cycle arrest (Qi et al., 2021), while on the other hand, inhibition of TRPML1 function has also been reported to impair autophagy induction and inhibit cancer cell invasion and migration (Rühl et al., 2021). Consequently, the role of TRPML1 in cancer is still subject to intensive investigations.

Interestingly, TRPML1 dysregulation has recently been associated with disrupting mitochondrial function. While Otto Warburg postulated over 60 years ago, that mitochondria in cancer cells are ‘broken’ (Warburg, 1956), they are now widely known to be essential in cancer cells, regulating, for example, metabolic reprograming, Ca^2+^ homeostasis, generation of reactive oxygen species (ROS) and apoptotic cell death (Lopez and Tait, 2015; Wallace, 2012). Interestingly, TRPML1 malfunction has been connected to defective Ca^2+^ uptake of mitochondria (Peng et al., 2020), while release of lysosomal Zn^2+^ by TRPML1 caused mitochondria-mediated cell death (Du et al., 2021). Hence, TRPML1 seems to have a balancing role in mitochondrial function and which we further investigated in hepatocellular carcinoma. In this study, we developed a model to study TRPML1 over-activation and loss of function in HCC and identified the channel as a regulator of mitochondrial function in cancer cells.

## RESULTS

### Generation of a cellular model to study TRPML1 function

**Fig. 1.**
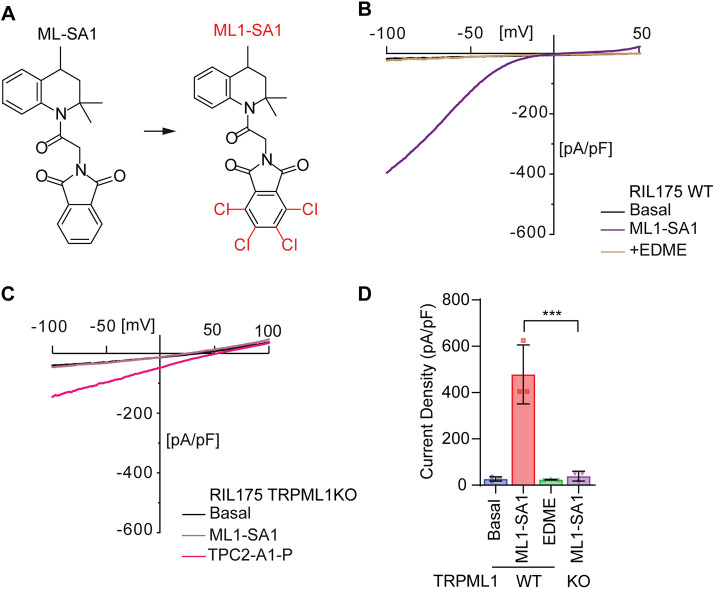
**Generation of a cellular model to study TRPML1 function.** (A) Chemical structures of MLSA-1 and ML1-SA1. (B,C) Representative I/V-plots of whole-endolysosome recordings from RIL175 WT (B) and TRPML1 KO (C) cells after treatment with ML1-SA1, TPC2-A1-P or EDME. TRPML1 inhibitor EDME (Rühl et al., 2021) and TPC2 activator TPC2-A1-P (Gerndt et al., 2020) served as experimental controls. (D) Statistical analysis of current densities evoked by treatment. Results represent mean±s.e.m. (*n*=3). ***P<0.005 (one-way ANOVA, followed by Tukey's post-hoc test).

To study TRPML1 function in HCC cells, we first started by establishing a model system in which we can activate or inhibit channel function. In the literature, some activators for TRPML1 have been described, such as MLSA-1, MLSA-5 and MK6-83. However, these activators lack specificity for TRPML channel subtypes (Grimm, 2016; Plesch et al., 2018; Shen et al., 2012). Recently, an isoform-selective activator for TRPML1, based on the structure of MLSA-1, called ML1-SA-1, has been developed by introduction of four additional chlorine substituents in the phthalimide subunit as characterized in Spix et al. (2022) (Fig. 1A). Hence, we utilized this isoform-selective TRPML1 activator to study the biological consequences of channel activation. Next, we used the CRISPR/Cas9 system to generate a TRPML1-knockout (KO) cell line for studying loss of channel function by deletion of exon 2 of the *MCOLN1* gene which encodes for the channel pore, as described previously (Bauer et al., 2015). Endolysosomal patch-clamp data obtained as described previously (Chen et al., 2017b) from TRPML1 wild-type (WT, Fig. 1B) and KO (Fig. 1C) cells showed the absence of TRPML1 currents after ML1-SA-1 activation (Fig. 1D) confirming a successful loss of function. Successful deletion of exon 2 was confirmed by PCR methods and Sanger sequencing (Fig. S1). In summary, we have generated a cellular model in which we can activate TRPML1 specifically and in which we have knocked out the channel (*MCOLN1*^−/−^) to study consequences of both overactivation and depletion.

### Functional characterization of cellular activation and KO models

**Fig. 2.**
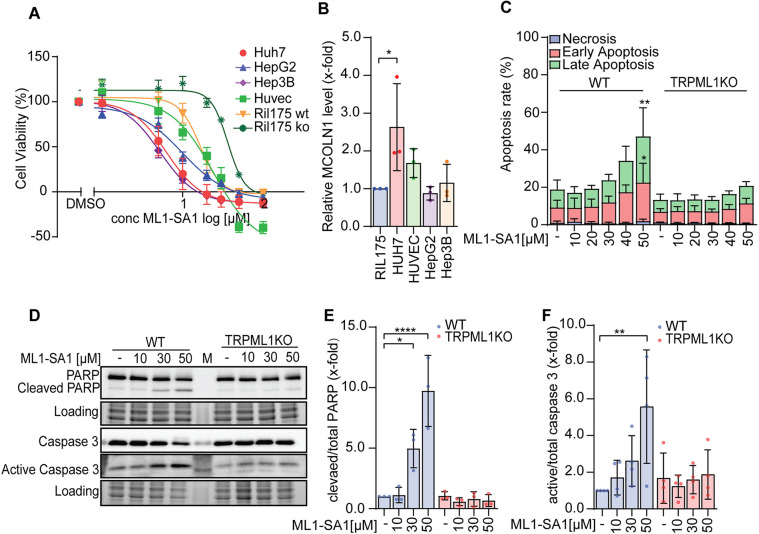
**Functional characterization of TRPML1 function in HCC cells.** (A) Proliferation after ML1-SA1 treatment was assessed with a CellTiter Blue^TM^ assay. (B) Relative mRNA levels of *MCOLN1* determined by qPCR in different cell lines. (C) Quantification of the percentage of apoptotic cells determined by Annexin V-PI staining and subsequent flow cytometry after 48 h treatment for the RIL175 WT and TRPML1 KO cells. (D) Western blot of PARP, caspase 3 and active caspase 3 protein level after 24 h treatment of for the RIL175 WT and TRPML1 KO cells. One representative image is shown. (E,F) Quantification of cleaved PARP/total PARP level (E) and active Caspase 3/total Caspase 3 ratios (F). Results represent mean±s.d. (*n*=3). *P<0.05, **P<0.01, ****P<0.0001 (two-way ANOVA for C–F and one-way ANOVA for B, followed by Tukey's post-hoc test). Results in B,E,F are shown relative to control set at 1.

Given that we are interested in analyzing the role of TRPML1 as target in anti-cancer therapy for HCC, we next investigated cell viability in our newly generated model. We determined IC_50_ values for ML1-SA1 in different HCC cell lines and the non-malignant human umbilical vein endothelial cells (HUVECs) (Fig. 2A), which express TRPML1 (Fig. 2B). IC_50_ values for ML1-SA1 are in the low micromolar range and are significantly higher in healthy HUVEC cells (Table 1). This indicates some degree of cancer cell specificity for the compound. Importantly, the IC_50_ for ML1-SA1 is significantly higher in TRPML1 KO cells (36.45 µM) as compared to TRPML1 WT cells (16.44 µM), underlining an on-target activity.

**Table 1.**
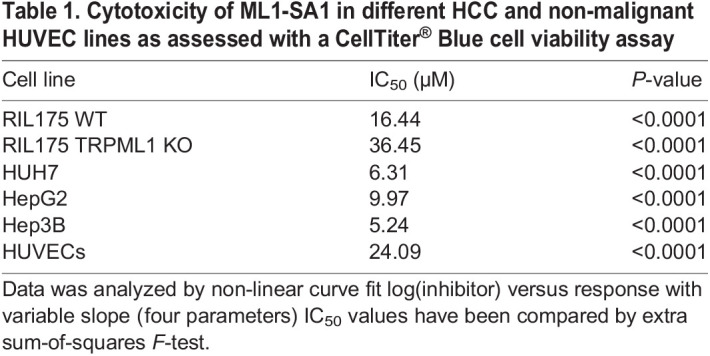
Cytotoxicity of ML1-SA1 in different HCC and non-malignant HUVEC lines as assessed with a CellTiter^®^ Blue cell viability assay

We next analyzed cell death induction by TRPML1 activation and KO (Fig. 2C) using Annexin V–FITC and propidium iodide (PI) staining and analysis by flow cytometry. Annexin V binds to phosphatidylserine residues, which are flipped to the outer surface of the membrane early in apoptosis. Co-staining with PI, which labels cellular DNA allows differentiation between early apoptosis (Annexin V positive and PI negative), late apoptosis (Annexin V positive and PI positive) and necrosis (Annexin V negative and PI positive) (van Engeland et al., 1998). Channel activation by ML1-SA1 significantly increased early and late apoptosis when at concentrations of 30 µM or higher. TRPML1 KO showed no influence on basal apoptosis levels. To exclude off-target cytotoxicity of ML1-SA1, we also analyzed apoptosis in TRMPL1 KO cells treated with 10–50 µM of ML1-SA1. ML1-SA1 did not induce apoptosis in these cells, indicating that apoptosis in wild-type cells is caused by TRPML1 activation and not by off-target cytotoxicity (Fig. 2C). Apoptosis induction could be confirmed by detection of PARP (herein referring to PARP1) cleavage and caspase 3 activation (Fig. 2D). Both classical apoptosis markers are significantly increased at concentrations of 30–50 µM ML1-SA1 (Fig. 2E,F). Along this line, TRPML1 KO had no effect on apoptosis marker expression levels and, given that these were also not changed upon ML1-SA1 treatment of TRPML1 KO cells, we conclude that TRPML1 activation is causative for apoptosis induction. Furthermore, we conclude that our tools and models are suitable to study the role of TRPML1 function in HCC cells.

### Proteomic analysis and electron microscopy reveal TRPML1 as regulator of mitochondrial morphology

**Fig. 3.**
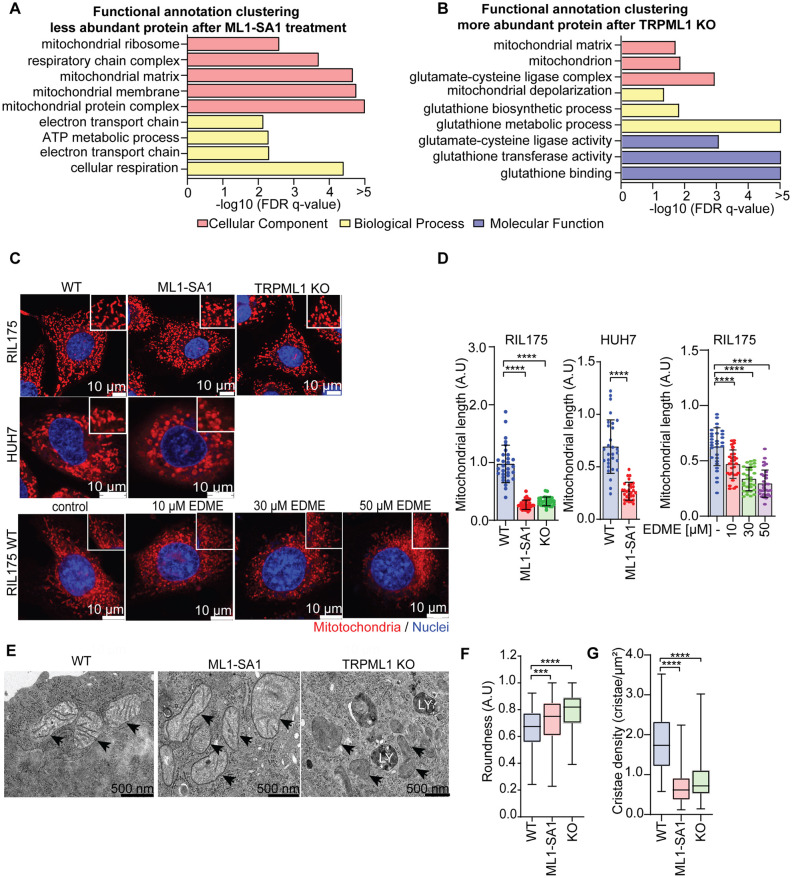
**TRPML1 regulates mitochondrial morphology.** (A,B) Proteomic analysis was performed by liquid chromatography tandem mass spectrometry (LC-MS/MS). Gene set enrichment analysis (GSEA) showed significant enriched gene sets of 30 µM ML1-SA1-treated or TRPML1 KO cells versus untreated RIL175 WT with FDR *q*-value ≤0.05. (A) STRING data analysis indicates downregulated protein clusters after ML1-SA1 treatment. (B) The STRING data analysis indicates upregulated protein clusters in TRPML1 KO cells. The gene ontologies of cellular component, biological process, and molecular function are coded in pink, yellow and purple, respectively. The *x*-axis shows the enrichment significance in −log10 (FDR *q*-value). (C) The mitochondrial morphology was observed by MitotrackerRed^TM^ (mitochondria) and Hoechst 33342 (nuclei) staining, using confocal live-cell imaging. Cells were treated as indicated with 30 µM ML1-SA1 or EDME, respectively. Scale bar: 10 µm. One representative image is shown. (D) Mitochondrial length of 30 cells from D per replicate was quantitatively measured by ImageJ. (E) Representative TEM images of RIL175 WT cells, 30 µM ML1-SA1-treated cells and RIL175 TRPML1 KO cells. Scale bar: 500 nm. (F,G) The roundness (F) and cristae density (G) (number of cristae/area of mitochondria) of WT, TRPML1 KO and ML1-SA1-treated RIL175 cells were quantified with ImageJ. A total of 150 mitochondria per replicate were measured in F and 100 mitochondria in G. Results represent mean±s.d. (*n*=5 for A,B; *n*=3 for C–G). ***P<0.005, ****P<0.0001 (one-way ANOVA, for D and G, followed by Dunnett′s post-hoc test; Kruskal–Wallis test for *F*, followed by Dunn′s post-hoc test). A.U, arbitrary units.

Having established a suitable cellular model, we aimed to elucidate cellular consequences triggered by modulating TRPML1 function. Therefore, we performed an analysis of the proteome in untreated RIL175 cells, as well as TRPML1 activated and TRPML1 KO cells. An unbiased quantitative proteome analysis showed a clear hierarchical clustering of the analyzed samples into the different analysis groups (Fig. S2). In total, 3219 proteins were identified (Fig. S2, Table S2) and a functional gene-set enrichment analysis between ML1-SA1 treated and untreated cells revealed that, predominantly, proteins responsible for mitochondrial structure are downregulated, while several regulatory circuits of other biological processes, such as ribosomal biogenesis, are upregulated (Fig. 3A; Table S3). Gene-set enrichment of TRPML1 KO cells also depicted a deregulation in mitochondrial regulatory circuits by trend, and a clear upregulation of glutathione metabolism (Fig. 3B; Tables S4–S6). These findings led us to further investigate morphology and function of mitochondria after modulating TRPML1 function. Confocal microscopy of mitochondria stained with MitoTracker^TM^ Red revealed that TRPML1 activation by ML1-SA1 led to a morphological shift from elongated mitochondria to short, round shaped structures in RIL175 as well as HUH7 cells (Fig. 3C,D). Interestingly, the same phenotype was also observed in TRPML1 KO cells and upon pharmacological TRPML1 inhibition with the specific inhibitor EDME (Rühl et al., 2021) (Fig. 3C,D). A more detailed analysis of mitochondrial morphology using transmission electron microscopy confirmed the switch of an elongated morphology in wild-type cells to short, round shaped mitochondria after modulating TRPML1 function (Fig. 3E). In particular, TRPML1 activation by ML1-SA1 led to a increase in mitochondrial roundness (Fig. 3F), which is associated with worsened mitochondrial function (Karbowski and Youle, 2003), and reduction in cristae density (Fig. 3G). These data clearly reveal that, upon altering TRPML1 function either by activation or KO, mitochondrial morphology is strongly altered.

### Modulating TRPML1 activity induces mitochondrial damage and dysfunction

**Fig. 4.**
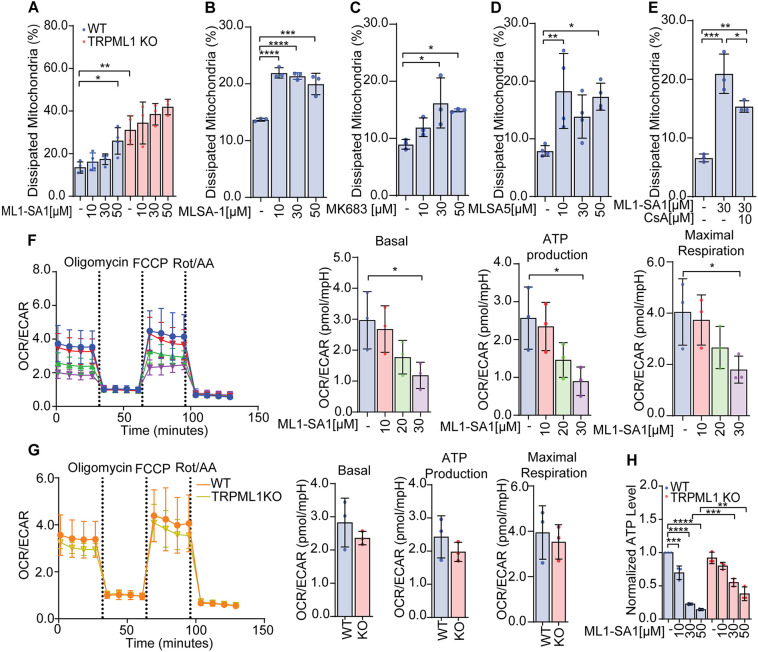
**TRPML1 activity regulates mitochondrial function.** The mitochondrial membrane potential of (A) RIL175 WT and RIL175 TRPML1 KO cells after 24 h treatment with ML1-SA1 as indicated was measured by flow cytometry after JC-1 staining. JC-1 staining of RIL175 WT cells after treatment with TRPML1 activators (B) ML-SA1, (C) MK6-83 and (D) MLSA5 was measured by flow cytometry. (E) Flow cytometry after JC-1 staining of RIL175 cells with or without pretreatment with the mPTP inhibitor cyclosporin A (CsA) for 2 h prior to 24 h incubation with ML1-SA1. Extracellular acidification rate (ECAR) and oxygen consumption rate (OCR) were measured in RIL175 cells in Mitostress test using a Seahorse 96XF analyzer. (F) RIL175 WT cells were pretreated with indicated concentration of ML1-SA1 for 24 h and the ATP production and maximal respiration of ML1-SA1 treated cells quantified by determining the OCR/ECAR ratio. (G) RIL175 WT and TRPML1 KO cells were compared. The Seahorse^TM^ measurement was normalized to cell number prior to calculation of the OCR/ECAR ratio. (H) ATP levels of RIL175 WT and TRPML1 KO cells, treated with ML1-SA1 were determined by CellTiter Glo^TM^ assay. Results represent mean±s.d. (*n*=3 or *n*=4 for D). *P<0.05, **P<0.01, ***P<0.005, ****P<0.0001 (one-way ANOVA followed by Dunnett's post-hoc test, Tukey's post-hoc for E and H, and Sidak's post-hoc test for A).

Since mitochondrial structure alterations might indicate mitochondrial functional deficits, we subsequently investigated mitochondrial integrity and respiratory capacity. To assess the damage of mitochondria, we measured the membrane potential by JC-1 staining. A shift from red to green fluorescence of JC-1 indicates a loss of membrane potential, depicting mitochondrial damage (Sivandzade et al., 2019). Activation of TRPML1 by ML1-SA1 led to a strong reduction in mitochondrial membrane potential, as did TRPML1 KO. ML1-SA1 treatment did not further increase mitochondrial membrane disruption in TRPML1 KO cells, confirming on-target activity of the compound (Fig. 4A). Of note, this effect could also be observed with other TRPML1 agonists, i.e. MLSA1 (Fig. 4B), MK6-83 (Fig. 4C) and MLSA5 (Fig. 4D). Depolarization of mitochondrial membranes is often mediated by formation and opening of the mitochondrial permeability transition pore (mPTP), which can be inhibited by cyclosporin A (CsA) (Mishra et al., 2019). Combination of ML1-SA1 with CsA could rescue mitochondrial depolarization, thereby confirming a loss of membrane potential mediated by mPTP activation (Fig. 4E). To determine the consequences of mitochondrial structural changes and loss of membrane polarity, we investigated the respiratory parameters by performing a Seahorse^TM^ mitochondrial stress test. In this test, cellular oxygen consumption is measured in real-time after stepwise addition of different inhibitors to probe mitochondrial function. First, oligomycin, an inhibitor of ATP synthase is added, which strongly reduces mitochondrial oxygen consumption and allows calculation of basal respiration. Secondly, the uncoupling agent FCCP is added, which collapses the proton gradient and induces maximal oxygen consumption. Finally, rotenone and antimycin A (Rot/AA) are added to inhibit mitochondrial function and enable determination of non-mitochondrial oxygen consumption. Pre-treatment of RIL175 cells with ML1-SA1 dose-dependently led to a reduction of basal respiration, ATP production and the maximal respiratory capacity of mitochondria (Fig. 4F; Fig. S3), confirming that mitochondrial changes induced by TRPML1 activation led to organelle dysfunction. In contrast, TRPML1 KO only led to a small decrease of basal respiration and ATP production, but did not decrease the maximal respiratory capacity, despite the structural changes we could observe (Fig. 4G; Fig. S3). We verified these findings by additionally analyzing cellular ATP levels using a luminescence-based ATP detection assay, which confirmed a significant reduction in cellular ATP upon ML1-SA1 treatment, but no significant change upon TRPML1 KO. Of note, ML1-SA1 showed no significant effect in TRPML1 KO cells (Fig. 4H). These data indicate that, even though mitochondria are structurally affected by both channel activation and loss of function in a similar way, functional consequences differ in quantity and quality.

### TRPML1 activation induces mitochondrial stress via Ca^2+^ overloading

**Fig. 5.**
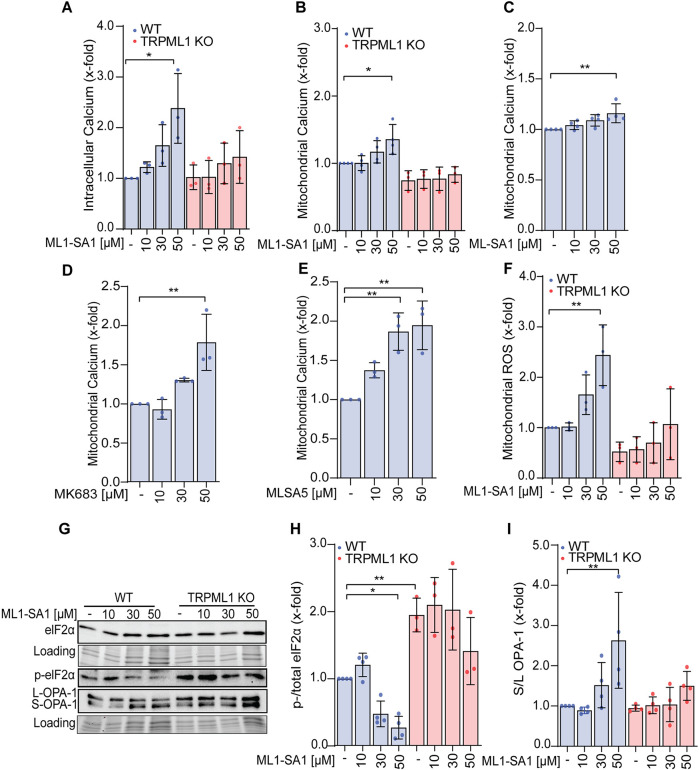
**TRPML1 activation induces mitochondrial stress via Ca^2+^ overloading.** (A,B) Intracellular (A) and mitochondrial Ca^2+^ (B) levels after stimulation with ML1-SA1 in RIL175 WT and RIL175 KO cells for 24 h were measured by means of Cal520 and Rhod-2 staining and flow cytometry, respectively. (C–E) Commercialized TRPML1 activators ML-SA1 (C), MK6-83 (D) and MLSA5 (E) were used for mitochondrial Ca^2+^ measurements in RIL175 WT cells by means of Rhod-2 and flow cytometry. (F) An oxidative stress in mitochondria of RIL175 WT and RIL175 KO cells after 24 h stimulation with ML1-SA1 was measured by MitoSox and flow cytometry. (G) Representative western blot of mitochondrial functional proteins after 24 h stimulation with ML1-SA1. (H,I) Quantification of (H) S/L-OPA1 and (I) p-/total elF2α. Results represent mean±s.d. (*n*=3; *n*=4 for B,C,H,I). *P<0.05, **P<0.01 (one-way ANOVA, followed by Dunnett's post-hoc test for C–E or Sidak's post-hoc test for A,F–I). Results in A–F,H,I are shown relative to control set at 1.

Following up on our findings, we aimed to investigate how channel activation or loss of function lead to functional changes in mitochondria on a mechanistic level. To begin with, we assessed the effects of TRPML1 activation on mitochondria in more detail. Since TRPML1 is a Ca^2+^ permeable ion channel, we measured intracellular Ca^2+^ levels by flow cytometry using a Ca^2+^-sensitive fluorescent dye. ML1-SA1 leads to a dose-dependent increase of intracellular Ca^2+^ levels, whereas KO of the channel has no effect and neither has ML1-SA1 treatment in KO cells (Fig. 5A). Furthermore, using Rhod2/AM, a Ca^2+^-sensitive fluorescent dye, which selectively locates to the mitochondria, we also showed that mitochondrial Ca^2+^ levels were increased after channel activation by ML1-SA1 in WT but not KO cells (Fig. 5B), with the same results seen upon MLSA1 (Fig. 5C), MK6-83 (Fig. 5D) and MLSA5 (Fig. 5E) treatment (Fig. 5B). A similar increase in mitochondrial Ca^2+^ levels was also observed in HUH7 cells (Fig. S4). As it is known that mitochondrial Ca^2+^ overload can lead to tremendous mitochondrial stress (Brookes et al., 2004), we assessed the levels of mitochondrial ROS. Along this line, treatment with ML1-SA1 led to an increase in mitochondrial ROS, while in TRPML1 KO cells, surprisingly, levels were even decreased and were not significantly altered by ML1-SA1 treatment (Fig. 5F). This somewhat puzzling effect might be explained by an upregulation of glutathione metabolism in TRPML1 KO cells, which was indicated in the proteome analysis (Fig. 3B). In agreement with these results, analysis of OPA1 showed a clear induction of protein cleavage [the level of long (L)-OPA-1 compared to short (S)-OPA1] in WT cells treated with ML1-SA1, but not KO cells (Fig. 5G,I). OPA1 is an inner mitochondrial membrane protein regulating mitochondrial fusion as well as cristae structure and contributes to ATP synthesis and apoptosis, which is typically cleaved during mitochondrial stress (Baker et al., 2014; MacVicar and Langer, 2016). Furthermore, eIF2a phosphorylation is decreased upon TRPML1 activation increased upon loss of channel function, and not further altered upon ML1-SA1 treatment in KO cells (Fig. 5G,H). As phosphorylation of this protein is a typical early stress response, which stabilizes mitochondrial function, this finding is in line with the observed mitochondrial dysfunction (Rajesh et al., 2013). Taken together, our data show that activating TRPML1 by means of ML1-SA1 leads to morphological and functional changes in the mitochondria and subsequent dysfunction caused by Ca^2+^ overloading, which induces mitochondrial stress and mPTP opening, which can be blocked by addition of CsA (Fig. 4E).

### Loss of TRPML1 function disturbs mitochondrial function by deregulation of mitophagy

**Fig. 6.**
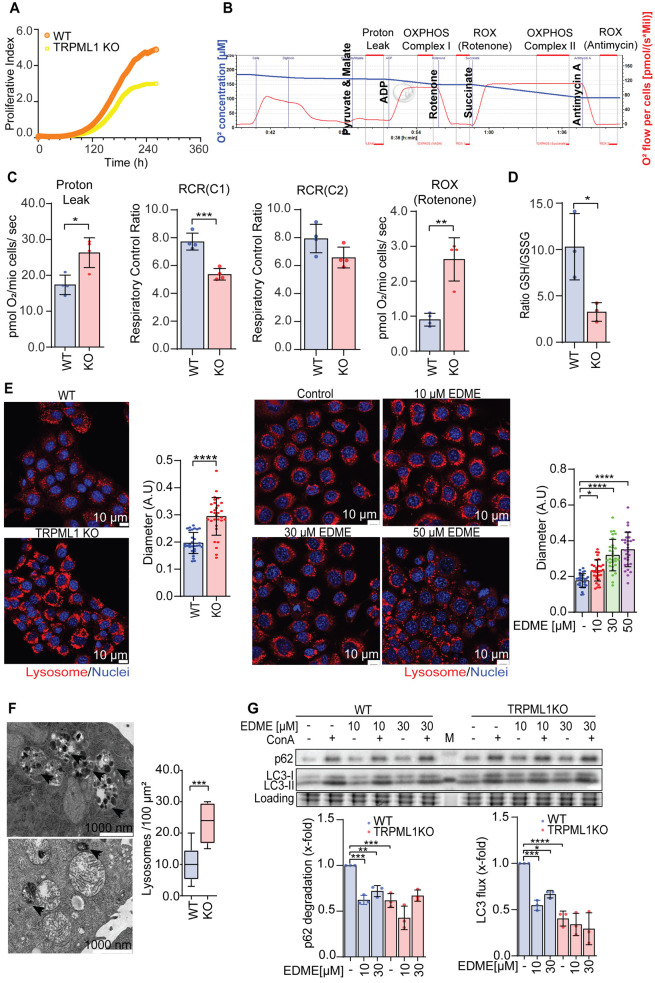
**Loss of TRPML1 function disturbs mitochondrial function.** (A) Proliferation of RIL175 WT and TRPML1 KO cells was determined by impedance measurements. (B) Schematic figure for mitochondrial respiration measurements with the Oroboros^®^ O2k. (C) Impairment of mitochondria in RIL175 KO cells was revealed by Oroboros^®^ O2K measurement. The respiratory control ratio (RCR) was calculated for indication about mitochondrial efficiency (OXPHOS/LEAK). The values were corrected for residual oxygen consumption (ROX of antimycin). (D) Ratio of GSH and GSSG between WT and TRPML1 KO cells (*n*=4). (E) Morphology of lysosomes in RIL175 WT, TRPML1 KO, and RIL175 WT cells after EDME treatment as indicated, was observed by Lysotracker Red staining and the diameter of lysosomes was measured by ImageJ (*n*=30). Scale bars: 10 µm. (F) Representative TEM images and quantification of number of lysosomes in WT and TRPML1 KO cells. Scale bars: 1000 nm. (G) Representative western blot of mitophagy markers in RIL175 WT and TRPML1 KO and quantification of LC3-II (lipidated LC3) flux and p62 degradation. Results represent mean±s.d. (*n*=3; or *n*=5 for C, *n*=4 for D). *P<0.05, **P<0.01, ***P<0.005, ****P<0.0001 [two-tailed unpaired Student's *t*-test (C,F); one-way ANOVA followed by Dunnett's post-hoc test (E) or Sidak's post-hoc test (G)]. Results in G are shown relative to control set at 1. A.U, arbitrary units.

Interestingly, we observed structural and functional changes in mitochondria upon TRPML1 activation and loss of function, respectively. However, the effects on mitochondrial function seen upon TRPML1 KO were not as pronounced as they were with channel activation (Figs 4 and 5) and the cells do not die from the KO (Fig. 2B). Nevertheless, mitochondrial function seems to be impaired in KO mitochondria as evidenced by a significantly elevated proton leak and, subsequently, proliferation of KO cells is slower than that of wild-type cells (Fig. 6A; Fig. S5A). A similar reduction in proliferation can be observed when pharmacologically blocking TRPML1 function (Fig. S5B,C). To assess how mitochondrial respiratory parameters are affected in more detail in TRPML1 KO cells, we measured functionality of different mitochondrial complexes by high-resolution respirometry using an Oroboros^®^ O2k-fluorespirometer. This allows a detailed analysis of the contribution of different mitochondrial complexes to mitochondrial respiration by subsequent addition of specific substrates and inhibitors (Fig. 6B). We found that in the KO cells, there is a significant proton leak (Fig. 6C; Fig. S5D), which agrees with the changes in mitochondrial structure and membrane depolarization we observed. We found that the respiratory control ratio (RCR) was significantly decreased in NADH-linked respiration (complex 1, C1) as well as succinate-linked respiration (complex 2, C2). This decrease in RCR is caused mainly by an increased proton leak (LEAK) in TRPML1 KO cells, since physiological respiration did not change significantly (Fig. 6C). Additionally, we found that non-mitochondrial respiration (residual oxygen consumption, ROX) after C1 inhibition with rotenone is increased (Fig. 6C). As this surprisingly did not increase mitochondrial ROS levels (Fig. 5F), but glutathione (GSH) metabolism is upregulated upon TRPML1 KO (Fig. 3B), we directly measured GSH levels. In the presence of ROS, GSH is oxidized to glutathione disulfide (GSSG) thereby detoxifying the cell from excessive ROS (Zitka et al., 2012). Indeed, we found that the ratio between GSH and GSSG is decreased (Fig. 6D), indicating that KO cells facilitate GSH to reduce ROS levels. Together with our Seahorse data (Fig. 4G), these findings suggest that TRPML1 KO leads to structural changes in mitochondria and a functional impairment, yet the cells can compensate for the loss and keep up respiration. Nevertheless, we wanted to analyze how loss of TRPML1 function leads to mitochondrial alterations, since it has been published that loss of TRPML1 impairs the autophagy and could subsequently lead to mitochondrial turnover. Along this line, we observed lysosomal swelling in KO cells, as well as upon pharmacological channel inhibition, compared to wild-type cells, by confocal microscopy (Fig. 6E) and transmission electron microscopy (Fig. 3E), without an increase in lysosomal mass (Fig. S5E). Quantitatively, the number of lysosomes is roughly doubled in the TRPML1 KO compared to WT cells, while on a qualitative basis the KO cells present more large lysosomal structures with membranous inclusions (Fig. 6F). Additionally, we detected the autophagic flux by assessing LC3 turnover and degradation of autophagic substrate p62 (also known as SQSTM1). A decrease of autophagic flux and degradation of p62 were observed in TRPML1 KO cells, as well as upon pharmacological inhibition of TRPML1 with the specific inhibitor EDME (Fig. 6G). Of note, EDME treatment of KO cells could not increase the effects, pointing to a TRMPL1-dependent impairment. These results suggest that disturbance of TRPML1 might disturb the renewal of mitochondria and lead to decrease of cancer cell proliferation.

## DISCUSSION

In this study, we reveal that the lysosomal cation channel TRPML1 acts as a regulator of mitochondrial function in hepatocellular carcinoma cells. Both overactivation of the channel by the TRPML1-isoform-selective activator ML1-SA1 as well as loss of function by genetic KO leads to a disturbance in mitochondrial function, yet of different extent and by different mechanisms.

Since Otto Warburg discovered aerobic glycolysis in cancer cells – a phenomenon now known as the ‘Warburg effect’ – it has become increasingly clear that mitochondria of cancer cells are altered. We now know that cancer cells reprogram their energy generation pathways and greatly depend on mitochondrial function, marking these organelles as interesting target in cancer therapy (Zong et al., 2016). Mitochondria not only generate energy, but can initiate apoptosis, generate ROS and regulate cellular Ca^2+^ homeostasis (Wallace, 2012). The Ca^2+^-permeable lysosomal TRPML1 channel has recently emerged as an important regulator of Ca^2+^ homeostasis and autophagic processes, rendering it also important for mitophagy (Huang et al., 2020; Scotto Rosato et al., 2019).

Ca^2+^ plays a central role in the structure, membrane potential and generation of reactive oxygen species (ROS) in mitochondria. Yet, the exact role of Ca^2+^ in mitophagy remains rather unclear (Rimessi et al., 2013). Given the importance of Ca^2+^ in mitophagy and mitochondrial regulation, it is likely that manipulating the Ca^2+^-permeable TRPML1 channel impacts mitochondrial function. Indeed, we found that activating TRPML1 increases cytosolic Ca^2+^ levels and further elevates mitochondrial Ca^2+^ levels (Fig. 5). This is in line with literature showing that mitochondria readily take up excessive Ca^2+^ from the cytosol (Duchen, 2000). Mitochondrial Ca^2+^ is important for the generation of ATP in these organelles (Hansford and Zorov, 1998; McCormack and Denton, 1993); nevertheless, Ca^2+^ overloading is dangerous. Excessive Ca^2+^ in the mitochondrial matrix can lead to enhanced generation of ROS, triggering the permeability transition pore, and cytochrome *c* release, leading to apoptosis (Brookes et al., 2004; Grijalba et al., 1999; Ott et al., 2002). Along this line, we detected a loss in mitochondrial membrane potential upon TRPML1 activation (Fig. 4A–D), which most likely resulted from opening of the mPTP, as potential loss could be prevented by CsA (Fig. 4E), a known blocker of mPTP formation (Brookes et al., 2004). Furthermore, we observed an increase in mitochondrial ROS production (Fig. 5F) and a loss of mitochondrial ATP production capacity (Fig. 4F,H) upon TRPML1 activation, strengthening our hypothesis. Since we also found an impairment of cell viability and an increase in apoptosis after TRPML1 activation (Fig. 2), we propose that overactivation of the channel leads to a release of Ca^2+^ into the cytosol. This could then be further amplified by Ca^2+^-induced Ca^2+^ release. Subsequently, mitochondria could take up the excessive Ca^2+^ and lose their structural integrity and membrane potential and produce ROS, which ultimately leads to cell death induction. In fact, our data are supported by a recently published study by Peng et al., who show that the lysosomal TRPML1 channel contributes to regulation of mitochondrial Ca^2+^ dynamics at lysosome–mitochondria contact sites (Peng et al., 2020). It is also worth mentioning, that TRPML1 is permeable for several cations, including Zn^2+^, which has been reported to induce cell death in melanoma cells upon pharmacological TRPML1 activation (Du et al., 2021).

While the role for TRPML1 activation in mitochondrial Ca^2+^ regulation becomes increasingly clear, the situation for a loss of channel function is more complex. Like overactivation of TRPML1, we observed that a KO of the channel in HCC cells leads to changes in mitochondrial morphology (Fig. 3); however, these changes do not lead to induction of cell death (Fig. 2). Furthermore, Ca^2+^ overloading of the mitochondria is unlikely to be the causal problem, as the Ca^2+^-permeable TRPML1 channel is not functional. We interestingly found in Seahorse^TM^ (Fig. 4G) and Oroboros^®^ O2k-fluorespirometric measurements (Fig. 6), that mitochondrial energy generation is only slightly impaired. However, we observed a somewhat puzzling decrease in mitochondrial membrane potential (Fig. 4A) and an increase in proton leak (Fig. 6C), indicating that there is indeed a mitochondrial impairment to some extent. As mentioned above, mitophagy and hence lysosomal function is essential for mitochondrial fitness. It has previously been reported that TRPML1 function is essential for lysosomal function (Medina et al., 2015; Settembre et al., 2012; Venkatachalam et al., 2015) and loss-of-function of the channel is implicated in lysosomal storage diseases, such as mucolipidosis type IV (Schmiege et al., 2018). In mucolipidosis type IV patients, lysosomes appear enlarged and swollen and, interestingly, deregulation in iron metabolism has been observed (Marques and Saftig, 2019; Schmiege et al., 2018). Along this line, we observed enlarged lysosomal structures by confocal and transmission electron microscopy (Fig. 3E), which suggest lysosomal impairment. This is a phenotype that has also been described for lysosomal storage diseases (Marques and Saftig, 2019).

Today, it is clear that autophagy is crucial for maintenance of cellular homeostasis. Cellular organelles usually undergo a constant cycle of degradation and renewal in which the organelle, or fragments thereof, are engulfed in autophagosomes and subsequently degraded in lysosomes and, afterwards, the organelle is renewed (Høyer-Hansen and Jäättelä, 2008; Mulcahy Levy and Thorburn, 2020). This process avoids organelle malfunction and can contribute to activity regulation. In mitophagy, the mitochondrial-selective form of autophagy, cellular stress conditions lead to ubiquitylation of mitochondrial outer membrane proteins, which are recognized by adaptor proteins like p62, and autophagosome formation is initiated through binding of LC3 family proteins (Palikaras et al., 2018). Our experiments show that TRPML1 loss of leads to blockage of autophagic flux (Fig. 6). This is in line with results in the literature reporting a regulatory role for TRPML1 in autophagy. In mucolipidosis type IV patients, macroautophagy has been reported to be disturbed, as indicated by accumulation of autophagic markers LC3 and p62 (Curcio-Morelli et al., 2010; Di Paola et al., 2018; Vergarajauregui et al., 2008). Furthermore, TRPML1-mediated Ca^2+^ release leads to activation of the phosphatase calcineurin, thus inducing TFEB nuclear translocation upon its dephosphorylation, and subsequent transcriptional activation of lysosomal and autophagic genes (Lieberman et al., 2012; Medina and Ballabio, 2015). Additionally, Scotto Rosato et al. recently reported TRPML1 is a multistep regulator of autophagy, which leads to generation of phosphatidylinositol 3-phosphate (PI3P) as well as the recruitment of PI3P-binding proteins, a process that requires the Ca^2+^-dependent kinase CaMKKβ and AMPK, which then induces formation of the ULK1 and VPS34 autophagic protein complexes (Scotto Rosato et al., 2019). Additionally, our data indicate an impairment of mitophagy upon TRPML1 malfunction. We found that KO cells show a roughly doubled number of lysosomes, which are enlarged and contain membranous incisions, probably being mitochondrial fragments (Fig. 6). An impairment of mitophagy has been associated with a number of pathophysiological conditions, including cancer. In these diseases, mitophagic disturbance was connected with increased ROS levels, altered cellular Ca^2+^ levels inflammation and apoptosis (Palikaras et al., 2018). Our data surprisingly revealed no increase in mitochondrial ROS levels (Fig. 5), which we propose is a consequence of an adapted glutathione (GSH) metabolism. Proteomic analysis and direct measurement showed an upregulation of GSH metabolism and an increased GSSG:GSH ratio in TRPML1 KO cells. It has been reported in that regard, that GSH is oxidized to glutathione disulfide (GSSG) in the presence of ROS, thereby detoxifying the cell and maintaining mitochondrial function (Zitka et al., 2012).

**Fig. 7.**
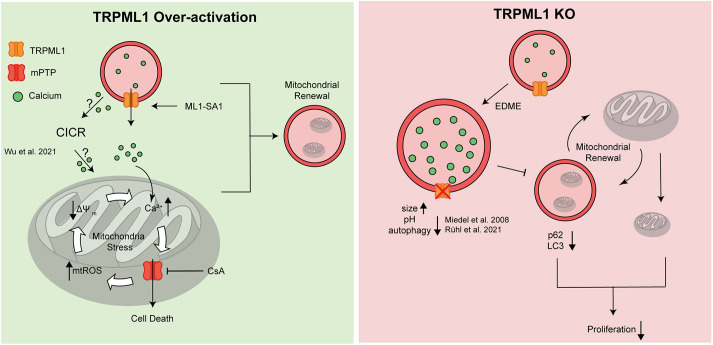
**Model of TRPML1 mitochondrial balancer function in HCC.** Left panel. In TRPML1 WT cells Ca^2+^ stored in the endolysosomal system can be released by the channel. Induction of TRPML1 overactivation by ML1-SA1 leads to Ca^2+^ release into the cytosol. This Ca^2+^ can either be taken up by mitochondria directly, or induce Ca^2+^-induced Ca^2+^ release from other organelles, for instance from the endoplasmic reticulum. Mitochondrial Ca^2+^ uptake causes mitochondrial membrane depolarization and generation of ROS. This leads to an opening of the mPTP and subsequent cell death, which can be prevented by CsA. Right panel. Upon TRPML1 KO, lysosomal function is impaired leading to a lysosomal swelling and impaired function. For instance, size as well as pH are altered. Subsequently, mitophagy and therefore mitochondrial renewal is disturbed. In summary, the impairment in mitochondrial and lysosomal function leads to a decreased cellular proliferation rate. mtROS, mitochondrial reactive oxygen species; ΔΨ_m_: mitochondrial membrane potential.

In summary, TRPML1 overexpression is associated with growth and survival of several cancer cells but has also been reported beneficial for other cancers. However, research has yet to identify the underlying mechanisms of these discrepancies (Morelli et al., 2019; Santoni et al., 2020). We report that in HCC cell lines, activation of TRPML1 function leads to cancer cell death by impairing mitochondrial function in a Ca^2+^-dependent manner and might therefore be beneficial in cancer therapy. On the other hand, we propose that TRPML1 loss-of-function leads to a disturbance in mitophagy, which, despite impairing mitochondrial function, does not induce cell death but instead leads to a reduced rate of proliferation (Fig. 7). Thereby we reveal a novel role for TRPML1 as a regulator of mitochondrial function, and its modulators as promising molecules for novel therapeutic options in HCC therapy. In conclusion, we propose that targeting TRPML1 might be a promising approach to target HCC by manipulating mitochondrial function.

## MATERIALS AND METHODS

### Cell lines

RIL175 WT cells were kindly provided by Prof. Simon Rothenfußer (Division of Clinical Pharmacology, University Hospital, LMU Munich, Germany) and had originally been isolated from hepatic tumors established in C57BL/6 mice as described previously (Müller et al., 2021). HUH7 cells were obtained from Japanese Collection of Research Bioresources (JCRB0403, Tokyo, Japan). HepG2 and Hep3B cells, and HUVECs were purchased from American Type Culture Collection (HB-8065, HB-8064, PCS-100-010 Manassas, Virginia, USA). Cancer cell lines were cultured in Dulbecco's modified Eagle's medium (DMEM; RIL175 WT and TRPML1 KO, HUH7, HepG2) or Eagle's minimum essential medium (EMEM) (Hep3B), supplemented with 10% fetal calf serum (FCS) and 100 U/ml penicillin-streptomycin (Pan Biotech, Aidenbach, Germany). The cells were maintained at 37°C in a humidified atmosphere of 5% CO_2_. None of the cell lines used are listed in the database of commonly misidentified cell lines maintained by ICLAC. All cells are proven to be mycoplasma-free on a quarterly basis.

### Compounds

The following compounds were used: the TRPML1 inhibitor, EDME (10–50 µM, synthesis as previously described; Rühl et al., 2021); TRPML1 activators, ML-SA1 (10–50 µM, SML0627, Sigma-Aldrich), MLSA5 (10–50 µM, kind gift from Casma Therapuetics, Cambridge, MA, USA) and MK683 (synthesized as described previously, Chen et al., 2014); and concanamycin A (ConA, 1 µM, Santa Cruz Biotechnology, Dallas, Texas, USA).

### CRISPR/Cas9-mediated KO generation

The KO of TRPML1 in murine RIL175 cells with the CRISPR-Cas9 system was conducted as described previously (Bauer et al., 2015; Ran et al., 2013). We deleted exon 2 of TRPML1 based on the *MCOLN1* sequence from ENSEMBL (https://www.ensembl.org/). Single-guide RNAs (sgRNAs) were designed as described previously (Haeussler et al., 2016). The sequence for the sgRNA upstream (5′) of exon 2 (5′-GTATACCCAAAATGTCCCCG-3′) was cloned into the eCas9_Puro2.0 plasmid using the T4 DNA ligase protocol as indicated by the manufacturer (New England Biolabs, Frankfurt am Main, Germany). The sequence for the sgRNA downstream (3′) of exon 2 (5′-GGGAAGGACGTAGGTAGATC-3′) was cloned into the eSpCas9_2A_Blasti plasmid (provided by Lars König, Klinikum der Universität München, Munich, Germany). The respective plasmids were transformed into competent DH5α *Escherichia coli* and afterwards prepared using the QIAGEN Plasmid Maxiprep Kit according to the manufacturer's instructions. Correct insertions were confirmed by Sanger sequencing starting from the U6 promoter. Subsequently, RIL175 WT cells were transfected using Lipofectamine^TM^ 3000 (Invitrogen Waltham, Massachusetts, USA) transfection reagent according to the manufacturer's instructions. Selection was performed with puromycin (2 µg/ml) and blasticidin S (6 µg/ml), respectively. Genomic DNA from potential KO colonies were amplified by PCR and analyzed by agarose gel electrophoresis. The DNA was extracted from the gel by the QIAquick gel extraction kit (#28704, QIAGEN, Düsseldorf, Germany) according to the manufacturer's instructions and analyzed by Sanger sequencing. We used a primer pair spanning the deleted region of exon 2 (forward: 5′-CCCACAGAAGAGGAAGACCT-3′, reverse: 5′-GCACAGTGACCACCAAGAT-3′) and a primer pair binding within the deleted region of exon 2 (forward: 5′-ACAGAATCCTAGACTGGCCT-3′, reverse: 5′-AAGGTGGGTACAGGAGTGGT-3′).

### Endolysosomal patch-clamp

For determination of TRPML channel function, whole endolysosomal patch-clamp recordings were conducted using a modified conventional patch clamp. In brief, endolysosomes of transfected HEK293 cells or RIL175 WT and KO cells were enlarged through treatment with 1 µM vacuolin (V7139, Sigma-Aldrich, St Louis, Missouri, USA) overnight. Intact endolysosomes were isolated and current recordings and data analysis were performed as described previously (Chen et al., 2017b). For optimal conditions for TRPML1 measurement, the luminal pH was adjusted to 4.6 and sodium methanesulfonate (Na-MSA; Sigma-Aldrich) was used in the luminal solution. For optimal conditions for TRPML2, the luminal pH was adjusted to 7.2 and Na-MSA was used in the luminal solution (Plesch et al., 2018). For optimal conditions for TRPML3, the luminal pH was adjusted to 7.2 and potassium methanesulfonate (K-MSA) was applied to replace Na-MSA in the luminal solution (Chen et al., 2017a). In all experiments, 500 ms voltage ramps from −100 to +100 mV were applied every 5 s, holding potential at 0 mV. The current amplitudes at −100 mV were extracted from individual ramp current recordings. Origin8 software was used for all statistical analysis.

### Proliferation assays

Cellular proliferation was assessed by means of a CellTiter-Blue^®^ assay and impedance measurement. CellTiter-Blue^®^ assay (Promega, Madison, Wisconsin, USA) was performed according to manufacturer's protocol after 72 h. Impedance measurements were performed using the xCELLigence RTCA device (ACEA Biosciences, San Diego, California, USA); the cell index, a dimensionless parameter that is proportional to the cell number, can be determined through impedance measurement. RIL175 WT and TRPML1 KO cells were seeded at a density of 2×10^3^ cells per well into an equilibrated 16-well E-plate. Slopes were calculated using the xCELLigence RTCA software (ACEA Biosciences, San Diego, California, USA) for each cell line until reaching the plateau phase using the following equation: Slope (1/h)=(Cell Index−intercept)/[time (h)].

### Quantitative real-time PCR

Isolation of mRNA (RNeasy Mini Kit # 74104, QIAGEN, Düsseldorf, Germany), synthesis of cDNA templates (High Capacity cDNA Reverse Transcription Kit # 4368814, Applied Biosystems, Foster City, California, USA) and quantitative real-time PCR (qPCR) (QuantStudio 3 Real-Time PCR system, Applied Biosystems) with actin serving as housekeeping gene were performed as described previously (Schneider et al., 2015). Primers were purchased from Metabion (Planegg, Germany) (Table S1).

### Flow cytometry

Cells were harvested from 24-well plates using trypsin/EDTA (PAN Biotech, Aidenbach, Germany) and transferred to tubes after the indicated treatment period. For apoptosis detection, harvesting was followed by staining with the eBioscience™ Annexin V-FITC Apoptosis Detection Kit (#BMS500FI-100, Invitrogen, Waltham, Massachusetts, USA) according to the manufacturer's protocol for the apoptosis test. Mitochondrial membrane potential (ΔΨm) was detected by means of JC-1 staining (#ENZ-52304, Enzo Life Science GmbH, Lörrach, Germany). Intracellular Ca^2+^ and mitochondrial Ca^2+^ were stained by means of Cal520 (ABD-21130, Biomol GmbH, Hamburg, Germany) and Rhod-2 (R1245MP, Thermo Fisher Scientific, Waltham, Massachusetts, USA), respectively. Cyclosporin A (#30024-25MG, Sigma-Aldrich) was applied for 2 h prior to indicated stimulation. At least 30,000 events were analyzed per sample by flow cytometry (Canto II, Beckton Dickinson, Franklin Lakes, New Jersey, USA).

### Western blotting

Cells were harvested and lysed in RIPA lysis buffer containing a protease inhibitor mix (#4693159001, Basel, Switzerland). Lysates were centrifuged at 10,000 ***g*** for 10 min and 4°C. Protein amounts were assessed by Bradford assay (#K015.1, Carl Roth, Karlsruhe, Germany), and an equal amount of protein was separated by SDS-PAGE and transferred to nitrocellulose membranes (Hybond-ECLTM, Amersham Bioscience, Buckinghamshire, UK). The membranes were incubated with blocking buffer [5% BSA in phosphate-buffered saline (PBS) with 0.1% Tween 20] for 1 h, followed by incubating with anti-PARP (1:1000, #9542, Cell Signaling, Danvers, Massachusetts, USA), anti-caspase 3 (1:1000, #sc-7148, Santa Cruz Biotechnology, Dallas, Texas, USA), anti-active caspase 3 (1:1000, #C8487, Sigma-Aldrich), anti-OPA-1 (1:1000, #80471, Cell Signaling), anti-elF2α (1:1000, #sc-133132, Santa Cruz Biotechnology), anti-phospho-elF2α (1:1000, #9721, Cell Signaling), anti-LC3 (1:1000, #4108, Cell Signaling), or anti-SQSTM1/p62 (1:1000, #5114, Cell Signaling) primary antibodies at 4°C overnight. Secondary antibodies were incubated accordingly for 1 h and conjugated with horseradish peroxidase (HRP) and freshly prepared ECL solution with 2.5 mM luminol afterwards. Conjugated proteins were detected with the ChemiDoc™ Touch Imaging System (Bio-Rad, Hercules, California, USA). The protein level was normalized with total protein level and quantified by Image Lab™ Software (Bio-Rad). The LC3 flux and p62 degradation levels were calculated as described previously (Zhang and Lin, 2016).

### Proteomic analysis

Cells were lysed in 8 M Urea/400 mM NH_4_HCO_3_ using an ultrasonic homogenizer with a cup resonator (Sonopuls GM3200, BR30, Bandelin, Berlin, Germany). Protein concentration was determined using a Bradford assay. 10 µg of total protein was reduced with dithioerythritol (5 mM final concentration) for 30 min at 37°C and alkylated for 30 min using iodoacetamide at a final concentration of 15 mM. The sample was diluted with water to give a final concentration of 1 M Urea and digested overnight at 37°C with modified porcine trypsin (Promega, Madison, Wisconsin, USA) at an enzyme:protein ratio of 1:50. For mass spectrometry analysis, an Ultimate 3000 nano-chromatography system (Thermo Fisher Scientific) coupled to Q Exactive HF X mass spectrometer (Thermo Fisher Scientific) was used. A total of 1 µg of peptides was injected and separated at 250 nl/min using 0.1% formic acid in water as solvent A and 0.1% formic acid in acetonitrile as solvent B. As separation column an Easy-Spray column (PepMap RSLC C18 2 μm 100 Å 75 μm×50 cm, Thermo Fisher Scientific) was used. The separation method consisted of a 160 min gradient from 3% to 25% solvent B and a 10 min gradient from 25% to 40% solvent B. MS and MS/MS spectra were acquired with a top 15 data-dependent method. For protein identification, MaxQuant (v.1.6.1.0) (Cox and Mann, 2008) and the murine subset of the UniProtKB/Swiss-Prot database was used.

For gene set enrichment analysis (GSEA), the identified proteomes of ML1-SA1 treated and corresponding control groups were imported together with fold change, followed by proteins with values/ranks analysis in the STRING database (STRING v11.5) (Szklarczyk et al., 2021). For STRING analysis, the identified proteomes of TRPML1 KO and WT cells were filtered based on *P*-value (*P*<0.05) and fold change (*x*≥0.6 or *x*≤−0.6), followed by multiple proteins analysis in the STRING database. Different gene ontologies were selected based on false discovery rate (*q*<0.05).

### Confocal microscopy

Cells were seeded on an eight-well µ-Slide (#80826, Ibidi, Gräfelfing, Germany) for 24 h and treated as indicated. The cells were rinsed with PBS and incubated with MitoTracker™ Red (100 nM, #M22425, Thermo Fisher Scientific) for 30 min or Lysotracker™ Red DND-99 (500 nM, #L7528, Thermo Fisher Scientific), followed by nuclei staining with Hoechst 33342 (2.5 µg/ml, H3570, Thermo Fisher Scientific) for 30 min. The slides were mounted with mounting buffer before covering by a coverslip. All images were observed by fluorescence microscopy (Leica SP8 Inverted Scanning Confocal Microscope, Wetzlar, Germany). The length of mitochondria and diameter of lysosomes were measured using the Fiji software (Schindelin et al., 2012).

### Transmission electron microscopy

Samples were subjected to electron microscopy with minor variations from Zischka et al. (2008). Briefly, 3×10^5^ cells were immediately pelleted, fixed in 2.5% glutaraldehyde, fixed with 1% osmium tetroxide, dehydrated with acetone, and embedded in EPON resin. Ultra-thin sections were stained with lead citrate and UranyLess and imaged on a 1200EX electron microscope (JEOL, Tokyo, Japan) at 60 kV. Electron micrographs were recorded with a KeenView II digital camera (Olympus, Munich, Germany) with iTEM Ver. 5.0 (analySIS FIVE, Olympus, Munich, Germany).

Mitochondrial roundness was quantified as described in Tobias et al. (2018) with ImageJ (V1.52i). Briefly, at least 150 mitochondria per condition were encircled with the polygonal section tool and fitted to an ellipse. Outliers were identified and removed with GraphPad Prism by the ROUT method at *Q*=1%. Mitochondrial cristae density was quantified by encircling 100 mitochondria per condition and manually counting the cristae, which were then divided by mitochondrial area. Lysosomes were counted manually in 100 µm^2^ rectangular sections, each composed from four 25 µm^2^ adjacent sections from the same slice. At least eight composite sections per condition were analyzed.

### Seahorse extracellular flux analysis

Assay medium was prepared from DMEM 5030 powder (Sigma-Aldrich) by supplementing with L-glutamine (4 mM) and NaCl (143 mM) and pH adjustment to 7.35±0.05 and subsequent sterile-filtration through a Millipore Express^®^ PLUS membrane filter (0.22 µM, Merck Millipore). Poly-D-lysine coating solution was prepared by diluting 5 mg poly-D-lysine hydrobromide (Sigma-Aldrich) in 50 ml sterile water. Prior to cell seeding, the XFe96 microplate (Agilent Technologies) was coated for 5 min at room temperature by adding 15 µl poly-D-lysine-coating solution per well. Poly-D-lysine coating solution was rinsed from the wells using sterile water, and the plate was dried for 2 h prior to adding collagen G coating solution (0.001% in PBS; Biochrom, Cambridge, UK). After incubation for 30 min at 37°C cells were seeded and allowed to adhere overnight. Cells were pre-treated for 24 h in culture medium, which was exchanged for assay medium 1 h prior to measurement. The Seahorse XFe96 sensor cartridge was hydrated according to the manufacturer's instructions. A Mitostress test was performed according to manufacturer's protocol on a Seahorse XFe96 Analyzer (Agilent Technologies). In brief, 1.5 µM oligomycin, 0.5 µM FCPP and 0.5 µM rotenone/antimycin A were automatically injected into the seahorse cartridge. Hoechst 33342 was simultaneously injected at the end of the test and cell density was subsequently measured by BioTek Cytation Cell Imaging Reader (Agilent Technologies). Data were analyzed with Wave 2.6.0 software (Agilent Technologies). For data evaluation, the oxygen consumption rate/extracellular acidification rate (OCR/ECAR) ratios were calculated as described previously (Zhang et al., 2012).

### Mitochondrial respiration

Mitochondrial respiration was measured in an Oxygraph-2k instrument (Oroboros Instruments, Innsbruck, Austria). Living cells (2×10^6^) were added to an equilibrated measuring chamber containing 2.0 ml Mir05 respiratory medium (0.5 mM EGTA, 3 mM MgCl_2_, 60 mM lactobionic acid, 20 mM taurine, 10 mM KH_2_PO_4_, 20 mM HEPES, 110 mM D-sucrose, 1 g/l BSA, pH 7.1). Cells were permeabilized by addition of digitonin, allowing for determination of LEAK respiration. Afterwards, pyruvate (5 mM), malate (2 mM) and ADP (2.5 mM) were added to determine NADH-linked respiration. Succinate-linked respiration was determined by subsequent addition of rotenone and succinate. Finally, residual oxygen consumption (ROX) was determined by addition of antimycin A. All substrates and inhibitors were used at the concentrations suggested by Oroboros Instruments. Data were analyzed with DatLab 7.4 (Oroboros Instruments).

### Cellular glutathione measurements

Cellular GSH and GSSG levels were determined according to Rahman et al. (2006) with slight modifications. Briefly, 0.3×10^6^ living cells were immediately mixed with an equal volume of 10% metaphosphoric acid (Sigma-Aldrich) and subsequently sonicated to precipitate proteins. Afterwards, samples were centrifuged (16,000 ***g*** for 2 min), and the supernatant was neutralized with triethanolamine. Samples for total GSH determination were measured without further processing, whereas samples for GSSG determination were treated with 2-vinylpyridine (Sigma-Aldrich) to mask GSH. The samples were added to GSH assay buffer (100 mM potassium phosphate, 0.3 mM NADPH, 20 U glutathione reductase, pH 7.4, Sigma-Aldrich). The reaction was initiated by adding 0.2 mM 5,5′-dithiobis-(2-nitrobenzoic acid) (DTNB, Sigma-Aldrich). GSH was measured through TNB formation at 412 nm and quantified using a GSH standard curve.

### Statistical analysis

All figures represent mean±s.d. of at least three independent experiments unless stated otherwise. Statistical differences for two groups were calculated using a two-tailed unpaired Student's *t*-test. For comparison of more than two groups, one- or two-way or Kruskal–Wallis analysis of variance (ANOVA) analysis was applied and followed by Tukey or Dunnett's post-hoc test. Graphs were made and calculations were performed with Prism 8 (San Diego, California, USA). *P<0.05, **P<0.01, ***P<0.005, and ****P<0.0001.

## Supplementary Material

Supplementary information

Reviewer comments
