# Supplementary material for: Lysosomal TRPML1 regulates mitochondrial function in hepatocellular carcinoma cells
Source: J Cell Sci. 2022 Mar 21;135(6):jcs259455. doi: 10.1242/jcs.259455 (PMC8977057; doi:10.1242/jcs.259455)
Supplement: Supplementary information [file joces-135-259455-s1.pdf]

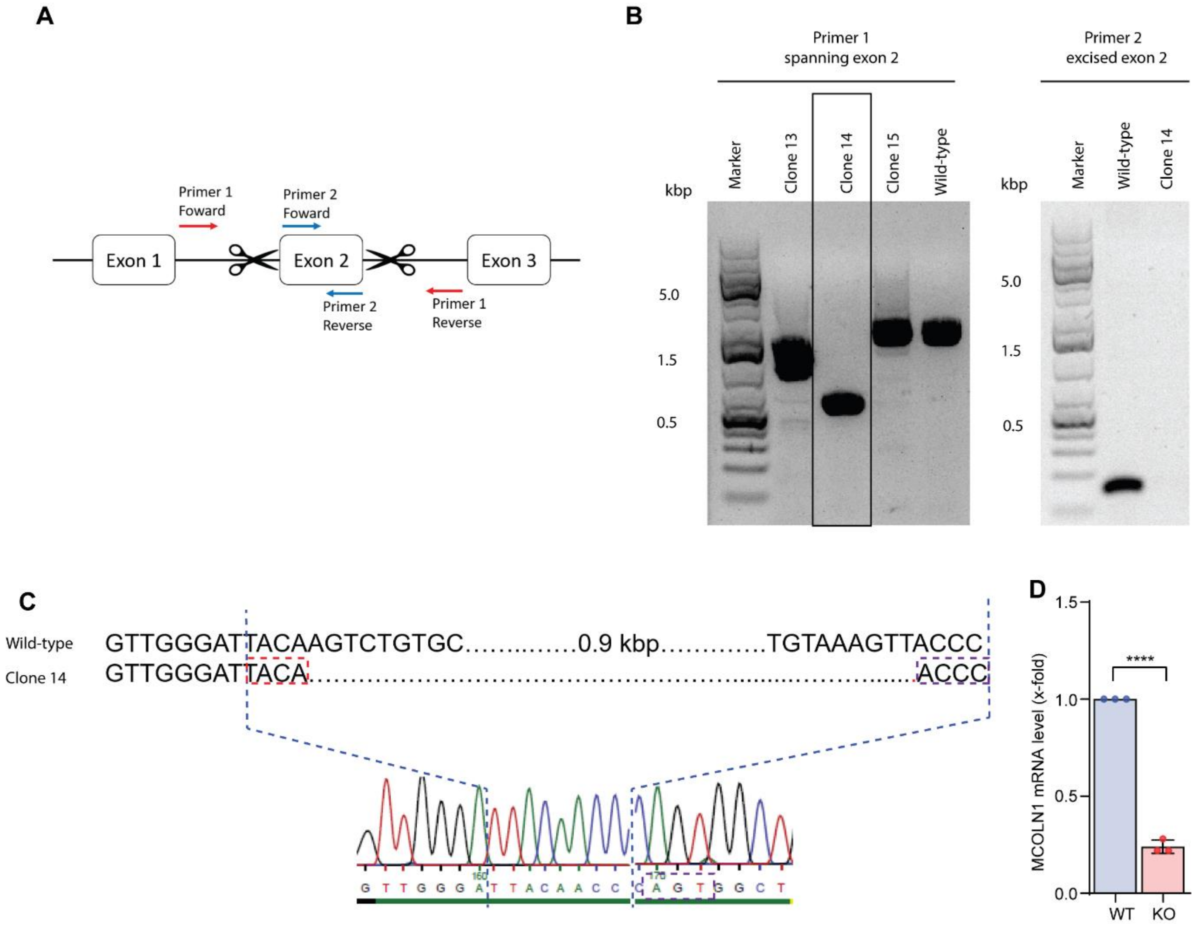


**Fig. S1. Generation of TRPML1 KO Clone**

(A) Strategy of creating TRPML1 KO clone. In brief, exon 2 (ex2) was the target for designated sgRNA (scissors). 2 pairs of primers were designed for validation. Primer 1 was detecting the spanning ex 2 and primer 2 was detecting the excised ex 2. (B) Agarose gel analysis indicated that clone 14 was the potential TRPML1 KO clone and subsequently validated by excised ex2 primer which binds within the ex2. (C) The clone 14 band on agarose gel was incised for Sanger sequencing. The deleted region was detected in clone 14 and residues base pair was ligated precisely. (D) The mRNA was extracted to performed qPCR. mRNA level of MCOLN1 was strongly reduced in clone 14, which indicate the presence of nonsense-mediated decay was occurred due to the deletion ex2. Results represent mean ± SD; n=3; unpair t-test; ****, p-value<0.0001.

Journal of Cell Science • Supplementary information

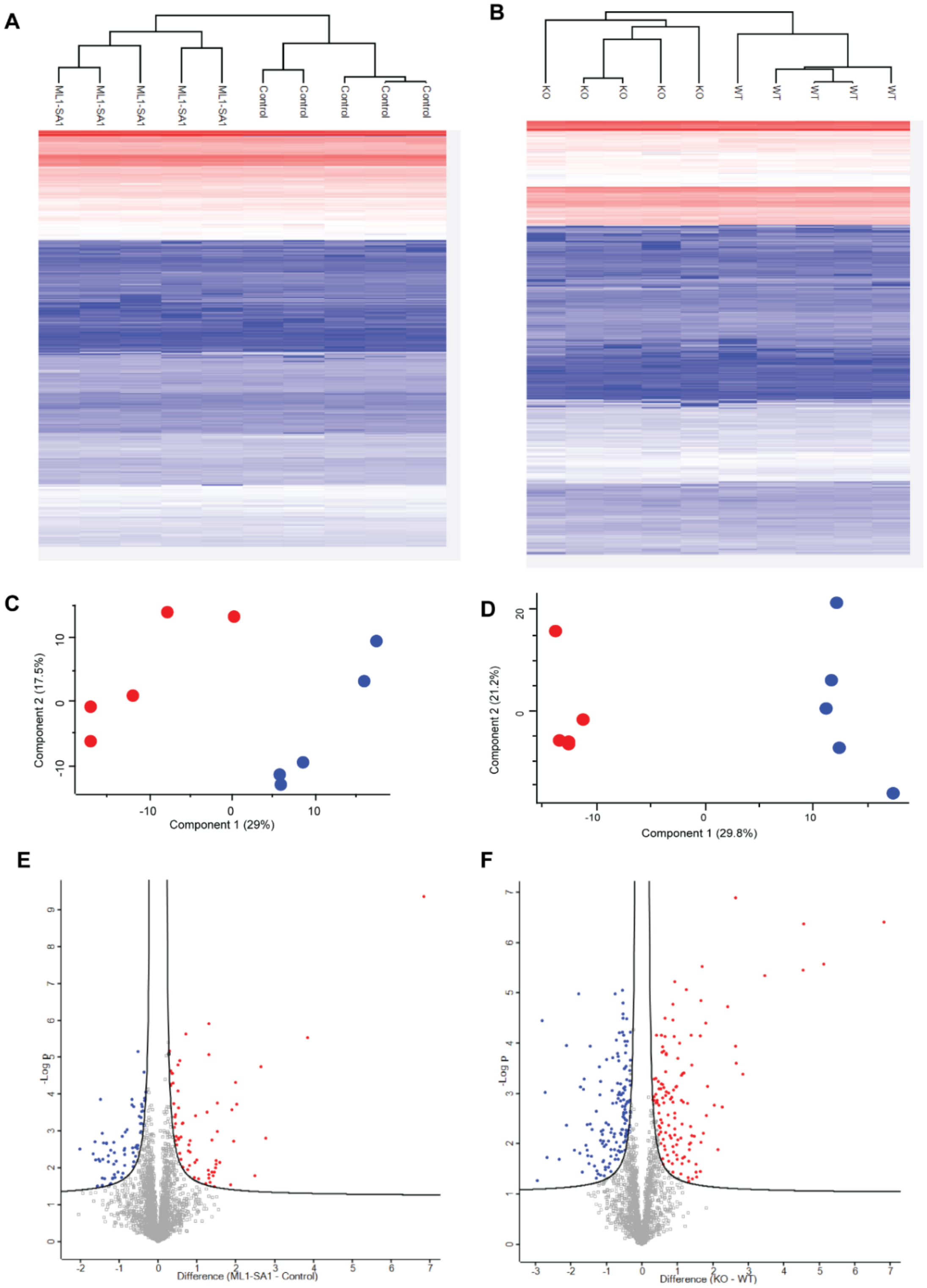


**Fig. S2. Proteome analysis of ML1-SA1 treated and TRPML1 KO cells**

(A, C, E) Aberration of the ML1-SA1 treated vs untreated RIL175 WT cell and (B, D, F) WT vs TRPML1 KO cell proteome. Both (A, B) heat map analysis and (C, D) principal component analysis (PCA) of iTRAQ intensity values clearly separate treated and untreated (A, C) or WT and KO (B, D) population. Volcano plot analysis of proteomes of (E) ML1-SA1 treated vs untreated and (F) WT vs KO indicate significant alterations of proteomes. Proteins differentially more abundant ($p < 0.05$) in ML1-SA1 treated or TRPML1 KO are highlighted in red, proteins less abundant in ML1-SA1 treated or TRPML1 KO are highlighted in blue. The full list of identified proteomes for ML1-SA1 treated and TRPML1 KO cells are listed in Table S2 and Table S4, respectively.

Journal of Cell Science • Supplementary information

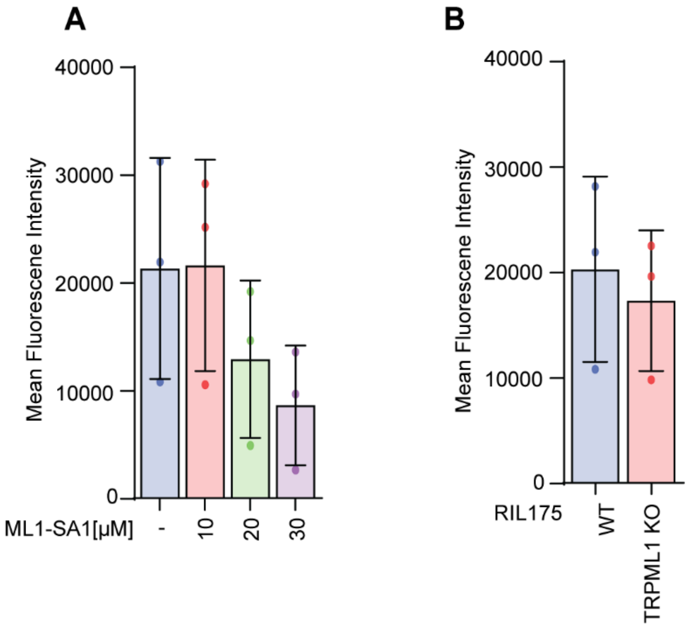


**Fig. S3. Normalized cell intensity of Seahorse assay in Figure 4F and G.**
Mean fluorescence intensity of (A) ML1-SA1 treated cell and (B) WT vs KO Seahorse experiment were performed after Mitostress measurement by Hoechst staining. All Mitostress test results were subsequently normalized to each cell intensity to avoid the artifact that caused by different cell densities. Results represent mean ± SD; n=3; Statistical analysis was performed one-way ANOVA followed by Dunnett´s post-hoc for A and unpaired t-test for B.

Journal of Cell Science • Supplementary information

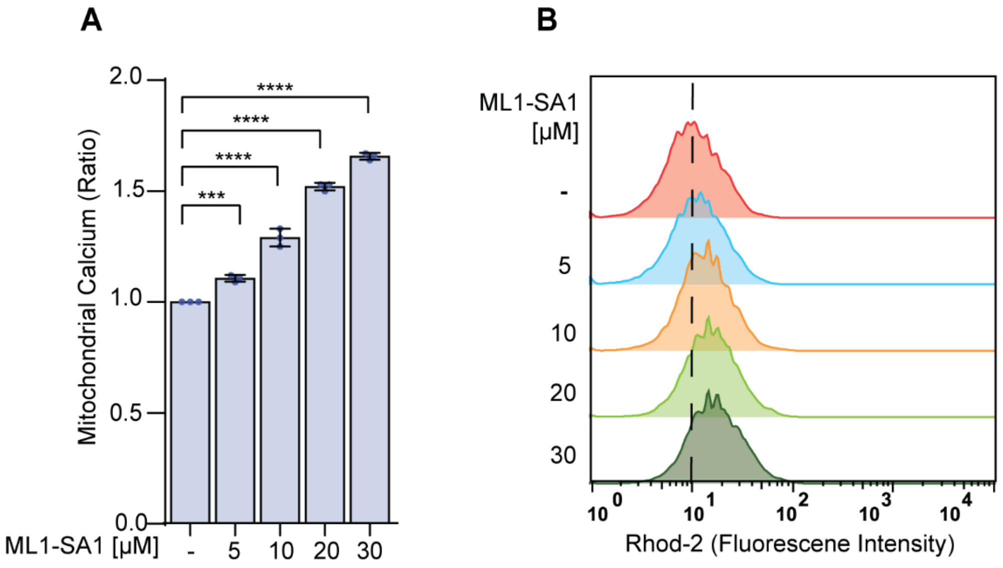


**Fig. S4. Mitochondrial calcium level after stimulated with ML1-SA1 in HUH7 cell line**

The ML1-SA1 were incubated with HUH7 cells for 24 h. (A) The Geometric Mean fluorescence intensity of ML1-SA1 treated HUH7 was normalized with control group. (B) Detected fluorescence changes of Rhod-2 was shown and black dash line indicated the basal line for comparison. Results represent mean ± SD; n=3; Statistical analysis was performed one-way ANOVA followed by Dunnett´s post-hoc for A and unpaired t-test for B. ***, p-value<0.0005, ****, p-value<0.0001.

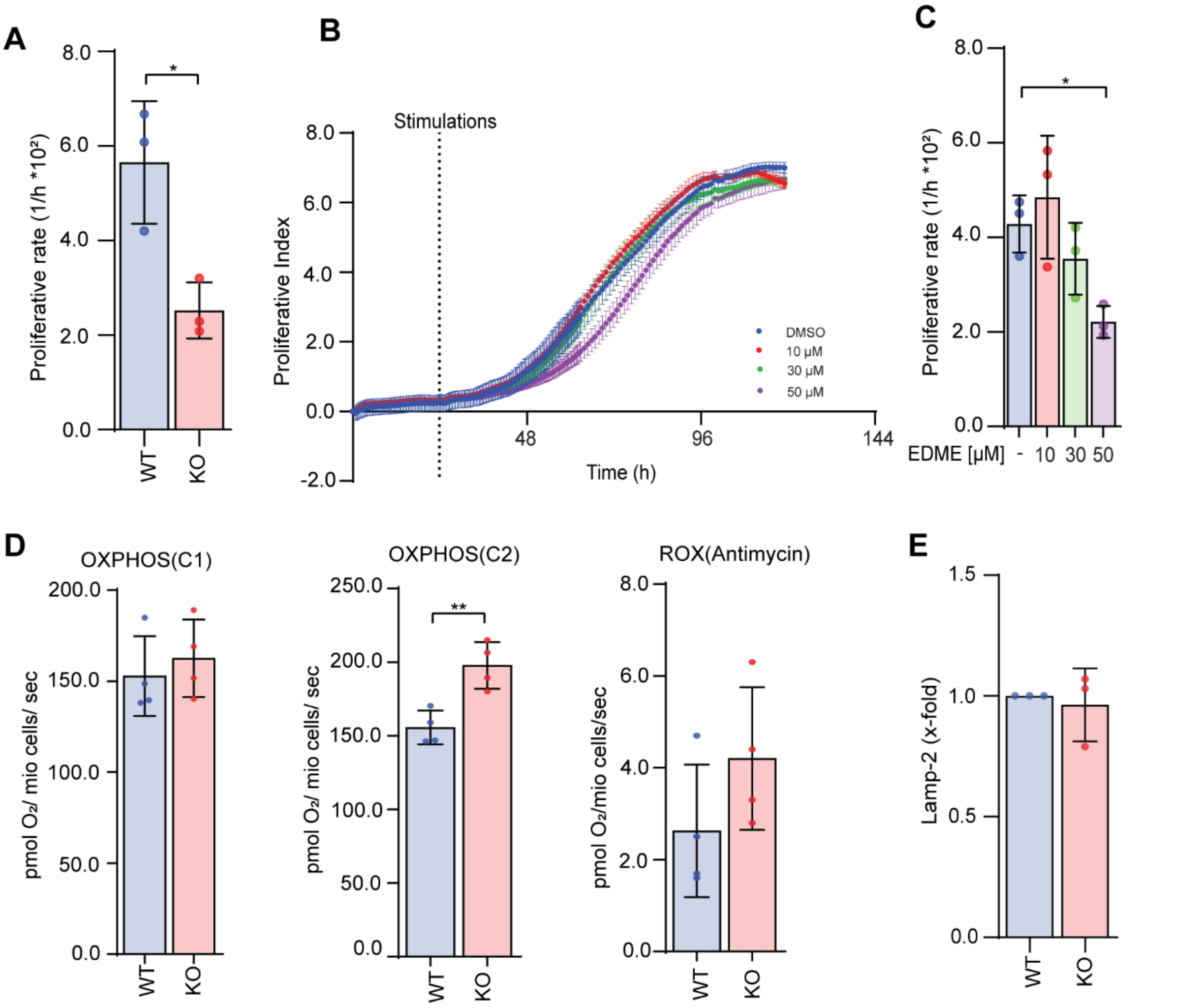


**Fig. S5. Loss of TRPML1 function disturbs mitochondrial function**

(A) Quantification of proliferative rate between RIL175 WT and TRPML1 KO cell. (B) Proliferation after EDME treatment was determined by impedance measurements. (C) Quantification of proliferative rate after the EDME treatment. (D) ROX of Antimycin treatment indicates the basal respiration of the cell. OXPHOS complex 1 and complex 2 results were deduced by ROX antimycin values. (E) Lysosomal mass was detected using anti-LAMP2 by western blot. Results represent mean ± SD; n=3; and n=4 for D. Statistical analysis was performed unpaired t-test. One-way ANOVA for C, followed by Dunnett's post-hoc test; *, p-value<0.05, **, p-value<0.01.

Journal of Cell Science • Supplementary information

**Table S1.** Table of primers

| Target genes | Forward primers (5´-3´) | Reverse primers (5´-3´) |
|---|---|---|
| h*MCOLN1* | TCTTCCAGCACGGAGACAAC | GCCACATGAACCCCACAAAC |
| m*MCOLN1* | GCCTTGGGCCAATGGATCA | CCCTTGGATCAATGTCAAAGGTA |
| h*Actin* | CCAACCGCGAGAAGATGA | CCAGAGGCGTACAGGGATAG |
| m*Actin* | CCACCATGTACCCAGGCATT | AGGGTGTAAAACGCAGCTCA |

**Table S2.** Proteins identified in the control (DMSO) and ML1-SA1 treated RIL175 WT cells.

Click here to download Table S2

**Table S3.** Gene set enrichment analysis of proteome from ML1-SA1 vs DMSO (Control) treated RIL175 WT. Enriched gene set in ML1-SA1 treated cells are listed in table A, while gene sets decreased in ML1-SA1 treated cells are listed in table B.

Click here to download Table S3

**Table S4.** Proteins identified in the RIL175 WT and MCOLN1 knockout (KO)cells.

Click here to download Table S4

**Table S5.** Proteins significantly different in abundance between RIL175 WT and MCOLN1 knockout (KO)cell. Positive log 2 fold changes indicates higher abundancy in KO cell. Negative log 2 fold changes indicates less abundancy in KO cell.

Click here to download Table S5

**Table S6.** STRING analysis of functional clustering between RIL175 WT and MCOLN1 KO cell (KO/WT). Annotated clusters for proteins that is more abundant in KO cell is listed in A, and less abundant are listed in B.

Click here to download Table S6

Journal of Cell Science • Supplementary information
