# Supplementary material for: Lysosomal TRPML1 regulates mitochondrial function in hepatocellular carcinoma cells
Source: J Cell Sci. 2022 Mar 21;135(6):jcs259455. doi: 10.1242/jcs.259455 (PMC8977057; doi:10.1242/jcs.259455)
Supplement: Reviewer comments [file joces-135-259455_review_history.pdf]

Journal of Cell Science

Lysosomal TRPML1 regulates mitochondrial function in hepatocellular carcinoma cells

Wei Xiong Siow, Yashar Kabiri, Rachel Tang, Yu-Kai Chao, Eva Plesch, Carola Eberhagen, Florian Flenkenthaler, Thomas Fröhlich, Franz Bracher, Christian Grimm, Martin Biel, Hans Zischka, Angelika M. Vollmar and Karin Bartel
DOI: 10.1242/jcs.259455

Editor: John Heath

Review timeline

Original submission:	16 November 2021
Editorial decision:	20 December 2021
First revision received:	10 January 2022
Accepted:	27 January 2022

Original submission

First decision letter

MS ID#: JOCES/2021/259455

MS TITLE: Lysosomal TRPML1 regulates mitochondrial function in hepatocellular carcinoma cells

AUTHORS: Wei-Xiong Siow, Yashar Kabiri, Rachel Tang, Yu-Kai Chao, Eva Plesch, Carola Eberhagen, Florian Flenkenthaler, Thomas Froehlich, Franz Bracher, Christian M Grimm, Martin Biel, Hans Zischka, Angelika M Vollmar, and Karin Bartel
ARTICLE TYPE: Research Article

We have now reached a decision on the above manuscript.

To see the reviewers' reports and a copy of this decision letter, please go to: https://submit-jcs.biologists.org and click on the 'Manuscripts with Decisions' queue in the Author Area. (Corresponding author only has access to reviews.)

As you will see, the reviewers raise a number of criticisms that prevent me from accepting the paper at this stage. They suggest, however, that a revised version might prove acceptable, if you can address their concerns. If you think that you can deal satisfactorily with the criticisms on revision, I would be pleased to see a revised manuscript. We would then return it to the reviewers.

We are aware that you may be experiencing disruption to the normal running of your lab that makes experimental revisions challenging. If it would be helpful, we encourage you to contact us to discuss your revision in greater detail. Please send us a point-by-point response indicating where you are able to address concerns raised (either experimentally or by changes to the text) and where you will not be able to do so within the normal timeframe of a revision. We will then provide further guidance. Please also note that we are happy to extend revision timeframes as necessary.

Please ensure that you clearly highlight all changes made in the revised manuscript. Please avoid using 'Tracked changes' in Word files as these are lost in PDF conversion.

© 2022. Published by The Company of Biologists under the terms of the Creative Commons Attribution License (https://creativecommons.org/licenses/by/4.0/).

I should be grateful if you would also provide a point-by-point response detailing how you have dealt with the points raised by the reviewers in the 'Response to Reviewers' box. Please attend to all of the reviewers' comments. If you do not agree with any of their criticisms or suggestions please explain clearly why this is so.

Reviewer 1

Advance summary and potential significance to field

The study investigated the role of the TRPML1 channel in the lysosome in the regulation of mitochondrial function in hepatocellular carcinoma (HCC) cells. The results showed pharmacological activation of TRPML1 and genetic depletion of its expression resulted in substantial structural and functional alterations in lysosome and particularly mitochondria, thereby impacting cell viability and proliferation.

Comments for the author

The study investigated the role of the TRPML1 channel in the lysosome in the regulation of mitochondrial function in hepatocellular carcinoma (HCC) cells.
The results showed pharmacological activation of TRPML1 and genetic depletion of its expression resulted in substantial structural and functional alterations in lysosome and particularly mitochondria, thereby impacting cell viability and proliferation. However, the study is not well or logically presented. Further experiments are required to provide a clear understanding of how the lysosomal TRPML1 mechanistically regulates mitochondrial function.

Major points:
1. The Introduction needs substantial revision to provide an updated understanding of the role of the TRPML1 expression, function and mechanisms in cancer cells. It misses the recent and highly relevant literature (e.g., Qi et al., 2021, Autophagy). The authors need to clearly formulate and present the research question.
2. The authors need to validate the expression of TRPML1, particularly its location to the lysosomes, and its role in mediating lysosomal calcium release.
3. On a related issue, the authors need to perform a better and full characterization of the TRPML1 expression in various types of cells examined using western blotting and patch-clamp recording. Such information may help them to clarify the differential sensitivity of different cell types to ML1-SA1 in affecting cell viability in Fig.2A-B, which is lack of a clear explanation.
4. Both ML1-SA1-induced TRMPL1 channel activation and depletion of TRPML1 expression resulted in similar effects on mitochondrial structure (Fig.3C-G) and mitochondrial membrane potential (Fig.4A), which are striking but how they are reconciled. In addition, the authors need to examine whether TRPML1-KO prevented ML1-SA1-induced effects on mitochondrial membrane potential (Fig.4A) and ATP production (Fig.4H).
5. Application of ML1-SA1 or other TRPML1 activators elevated intracellular calcium and mitochondrial calcium (Fig.5A-E). It is imperative to know whether TRPML1-KO abolished such calcium responses. Similarly, experiments are required to examine whether TRPML1-KO prevented ML1-SA1-induced mitochondrial ROS production (Fig.5F), and mitochondrial stress indicated by increased OPA1cleavage and reduced elF2α phosphorylation (Fig.5G-I).
6. The results summarized in Fig.6 clearly indicate that genetic depletion of TRPML1 expression introduced considerable alterations in both lysosomal and mitochondrial functions. Further experiments are required, for example, using TRPML1 inhibitors, to clarify how these effects due to TRPML1-KO is dependent or independent of the TRPML1 channel activity.
7. The mechanisms summarized in Fig.7 are not fully supported by the evidence from this study. For example, no evidence supports mitochondrial calcium is from calcium released from lysosome; mitochondrial calcium triggers opening of the mitochondrial transition pore; loss of mitochondrial membrane potential further increases mitochondrial calcium (left panel); and how TRPML1-KO causes lysosomal dysfunction (right panel).
8. The rationale of the experiments is not compelling and the way of presenting the data is poor in logic. It would be more logical and much easier to follow if the results are presented for example in an order: TRPML1 expression and function; effects of TRPML1 activation on lysosomal and mitochondrial structure and function, and cell viability and proliferation; effects of TRPML1-KO or

© 2022. Published by The Company of Biologists under the terms of the Creative Commons Attribution License (https://creativecommons.org/licenses/by/4.0/).

TRPML1 inhibitors on lysosomal and mitochondrial structure and function, and cell viability and proliferation; and, finally, TRPML1-KO or TRPML-1 inhibitors on TRML1 activator-induced effects on lysosomal and mitochondrial structure and function, and cell viability and proliferation
9. The Discussion session is a bit lengthy and speculative. More efforts are required to provide a clear interpretation of the results, and the significance of the finding from this study.

Minor points:
1. Describe the methods used in the order they appear in the Results session.
2. Provide the details of how to determine early and late apoptosis and necrosis in Fig.2C.
3. The results in Fig.3A-B are limited in significance and better placed with related supplemental data.
4. The writing needs substantial improvement in language expression and data presentation, for example, comparison of the data with to the literature should be in the Discussion rather session than the Results session!

Reviewer 2

Advance summary and potential significance to field

Summary of study

Liver cancers, including hepatocellular carcinoma (HCC), are the second most lethal cancers worldwide and novel therapeutic strategies are still highly needed.
Recently, the endolysosomal cation channel TRPML1 has gained focus in cancer research representing an interesting novel target.
The study utilized the recently developed isoform-selective TRPML1 activator ML1-SA1 and the CRISPR/Cas9 system to generate tools for over-activation and loss-of-function studies on TRPML1 in HCC.
The findings indicate that even though mitochondria are structurally affected by both channel activation and loss of function in a similar fashion, the functional consequences differ in both quantity and quality.
The study suggests that activating TRPML1 by ML1-SA1 leads to morphological and functional changes in the mitochondria and subsequent dysfunction, caused by calcium overloading, which induces mitochondrial stress and mPTP opening.
These results suggest that disturbance of TRPML1 might disturb the renewal of mitochondria and leads to decrease of cancer cell proliferation.
In summary, the findings of the study suggests that targeting TRPML1 might be a promising approach to target HCC by manipulating mitochondrial function.

My comments:
The study has produced some original and interesting findings of relevance to out understanding of cell biology and cancer therapy. The study is well executed and the report of the findings is well written and clear. The discussion makes some interesting points about the significance of the findings.

Comments for the author

I am happy with the manuscript and the findings of the study.
The methodology is clear and the study well executed.
The manuscript is well written.

© 2022. Published by The Company of Biologists under the terms of the Creative Commons Attribution License (https://creativecommons.org/licenses/by/4.0/).

First revision

Author response to reviewers' comments

Reviewer 1

Reviewer 1 Advance Summary and Potential Significance to Field:
The study investigated the role of the TRPML1 channel in the lysosome in the regulation of mitochondrial function in hepatocellular carcinoma (HCC) cells. The results showed pharmacological activation of TRPML1 and genetic depletion of its expression resulted in substantial structural and functional alterations in lysosome and particularly mitochondria, thereby impacting cell viability and proliferation.

Reviewer 1 Comments for the Author:
The study investigated the role of the TRPML1 channel in the lysosome in the regulation of mitochondrial function in hepatocellular carcinoma (HCC) cells. The results showed pharmacological activation of TRPML1 and genetic depletion of its expression resulted in substantial structural and functional alterations in lysosome and particularly mitochondria, thereby impacting cell viability and proliferation. However, the study is not well or logically presented. Further experiments are required to provide a clear understanding of how the lysosomal TRPML1 mechanistically regulates mitochondrial function.

Major points:

1. The Introduction needs substantial revision to provide an updated understanding of the role of the TRPML1 expression, function and mechanisms in cancer cells. It misses the recent and highly relevant literature (e.g., Qi et al., 2021, Autophagy). The authors need to clearly formulate and present the research question.

We thank the reviewer for pointing this out. We have adapted the introduction and included the suggested and further literature on this point. The changes have been highlighted in the revised manuscript.

2. The authors need to validate the expression of TRPML1, particularly its location to the lysosomes, and its role in mediating lysosomal calcium release.

We thank you for your comment. We totally agree with you, that normally, you should validate the KO also on a protein level. Unfortunately, the available antibodies for the TRPML1 channel do not produce a reliable band in a Western Blot experiment. We've added an example here to show you, that we don't get a reliable band on the membrane:

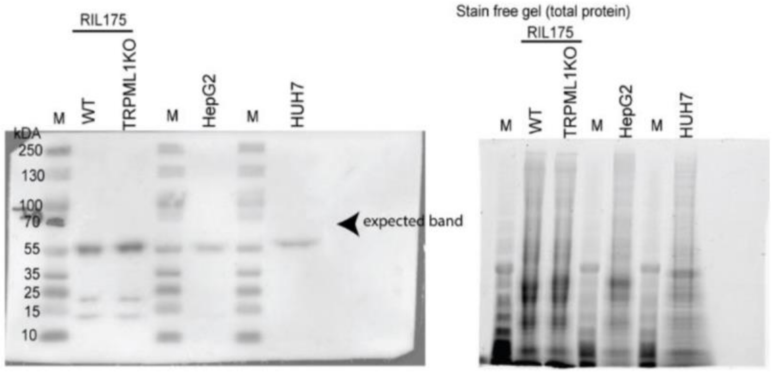

In this example, we've tested several cell lines with the TRPML1 antibody. Expected size of the band should be 65kDa according to the manufacturer. However, we don't see a band at this size, but rather several smaller bands. We also concluded that the 55kDa band visible here is not the TRPML1 band, as it does not vanish in the KO cells (which have been verified in several other ways). This was also true for several other cell lines tested, e.g. MEFs from validated and published TRPML1 KO mice. We've also tried using different antibodies or lysosomal fractions to obtain a better result.

© 2022. Published by The Company of Biologists under the terms of the Creative Commons Attribution License (https://creativecommons.org/licenses/by/4.0/).

Yet, we did not achieve a reliable result in any attempt. Therefore, we've decided to exclude Western Blotting for validation. However, we've verified the TRPML1 KO in other ways. As you can see from Figure S1 (which I've included here for your convenience), our strategy was to cut out exon 2 (Fig S1A) of the gene. DNA agarose gels showed a reduced size as a result of primer pair 1 amplification in our KO clone, which resembled the loss of exon 2. Further, primer pair 2, which binds within the exon 2 sequence did not give a band in the KO clone (Fig S1B). Moreover, Sanger sequencing of the respective area confirmed loss of exon 2 (Fig S1C) and mRNA levels of TRPML1 detected by qPCR were also down (Fig S1D). These data clearly confirm the loss of exon 2 in the KO cells.

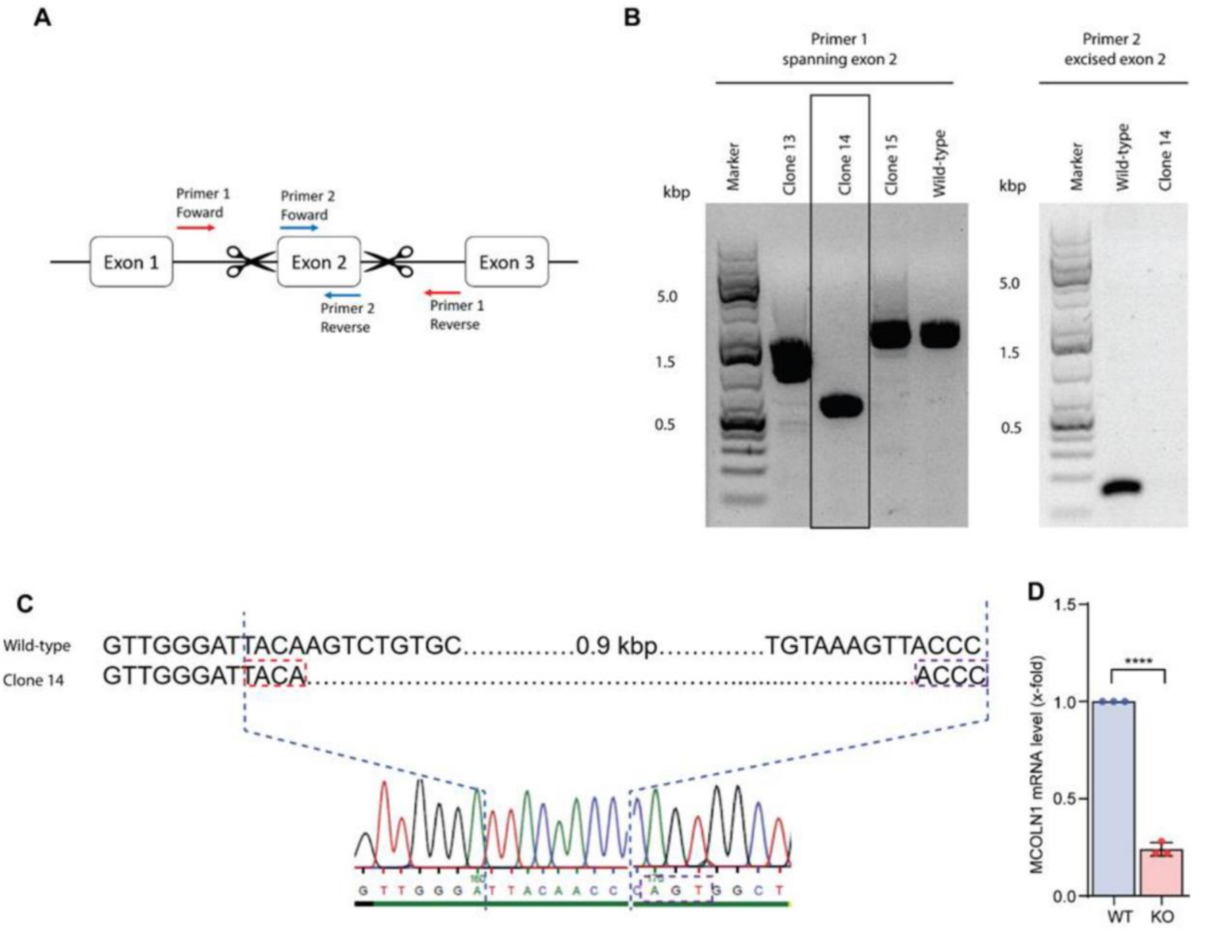

Figure S1 Generation of TRPML1 KO Clone
(A) Strategy of creating TRPML1 KO clone. In brief, exon 2 (ex2) was the target for designated sgRNA (scissors). 2 pairs of primers were designed for validation. Primer 1 was detecting the spanning ex 2 and primer 2 was detecting the excised ex 2. (B) Agarose gel analysis indicated that clone 14 was the potential TRPML1 KO clone and subsequently validated by excised ex2 primer which binds within the ex2. (C) The clone 14 band on agarose gel was incised for Sanger sequencing. The deleted region was detected in clone 14 and residues base pair was ligated precisely. (D) The mRNA was extracted to performed qPCR. mRNA level of MCOLN1 was strongly reduced in clone 14, which indicate the presence of nonsense-mediated decay was occurred due to the deletion ex2. Results represent mean ± SD; n=3; unpair t-test; ****, p-value<0.0001.

Additionally, we tested functional activity of TRPML1 in endolysosomal patch-clamp experiments, which are depicted in figure 1 of the main text (which I've additionally added for your convenience here).

© 2022. Published by The Company of Biologists under the terms of the Creative Commons Attribution License (https://creativecommons.org/licenses/by/4.0/).

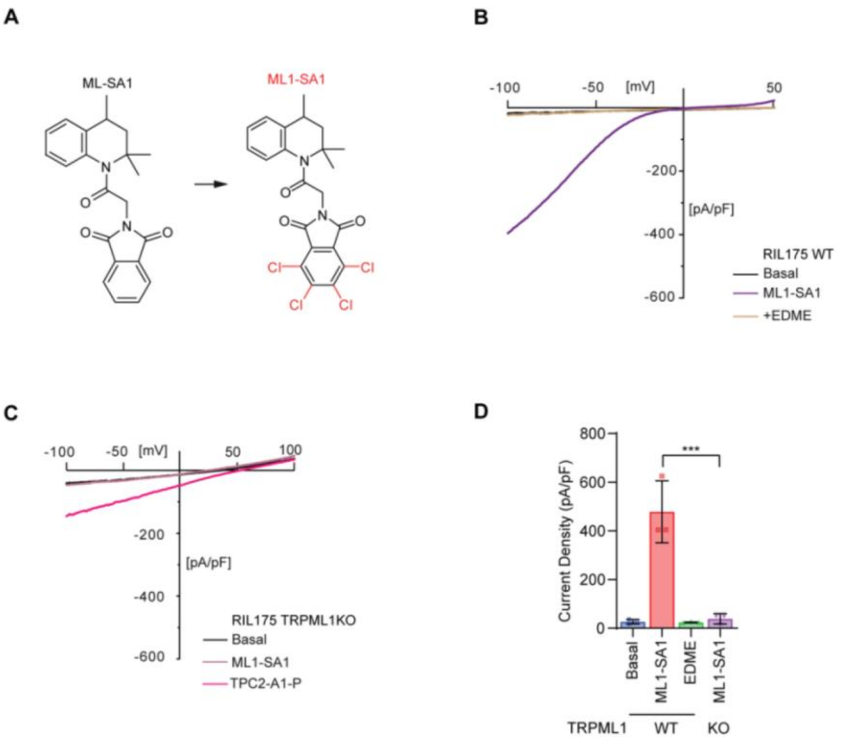

Figure 1. Generation of a cellular model to study TRPML1 function (A) Chemical structures of MLSA-1 and ML1- SA1. (B, C) Representative I/V-plots of whole-endolysosome recordings from RIL175 WT (B) and TRPML1 KO (C) cells after treatment with ML1-SA1, TPC2-A1-P or EDME. TRPML1 inhibitor EDME (Rühl et al., 2021)and TPC2 activator TPC2-A1-P (Gerndt et al., 2020) served as experimental controls. (D) Statistical analysis of current densities evoked by treatment. Results represent mean ± SEM; n =3; One-way ANOVA, followed by Tukey's post- hoc test ***, p-value <0.005, and ****, p-value <0.0001.

In this technique, channel currents from isolated lysosomes are recorded upon activation or inhibition with specific small molecules. As you can see, in WT cells TRPML1 current can be prominently recorded upon ML1-SA1 (Fig 1A) activation. It is further clearly inhibited by the specific inhibitor EDME (Fig 1B). In KO cells ML1-SA1 cannot induce a TRPML1 current, while TPC2-A1-P, a specific small molecule activator of TPC2, can still activate TPC2 currents (Fig 1C). This means, that we've obtained functional lysosomes in the KO cells, which do not depict a TRPML1 current, thereby confirming loss of the channel. Quantification of the data can be found in figure 1D.
In summary, we are sure, that we've created a successful TRPML1 KO, even though verification on Western Blot level was not possible.

3. On a related issue, the authors need to perform a better and full characterization of the TRPML1 expression in various types of cells examined, using western blotting and patch-clamp recording. Such information may help them to clarify the differential sensitivity of different cell types to ML1-SA1 in affecting cell viability in Fig.2A-B, which is lack of a clear explanation.

We thank the reviewer for this remark. However, a more detailed evaluation of TRPML1 expression in terms of protein expression and localization is not possible, due to a lack of specific antibodies, as described above. Unfortunately, this also makes confocal imaging regarding TRPML1 location highly difficult. Due to creating possible artefacts when using overexpressing constructs, we have decided not to use this approach. However, we do not doubt the lysosomal expression of TRPML1. It has been studied intensively in literature and there are hence many publications available, which show lysosomal localization of TRPML1. An overview can be found in Venkatachalam et al. (Venkatachalam et al., 2015) .Furthermore, TRPML1 specific currents can be detected in endolysosomal patch-clamp experiments. In this technique, which has been described in detail in (Chen et al., 2017a; Chen et al., 2017b), endolysosomes are enlarged and isolated from the cells.

© 2022. Published by The Company of Biologists under the terms of the Creative Commons Attribution License (https://creativecommons.org/licenses/by/4.0/).

After that, they are subjected to patch-clamp experiments, using endogenous, as well as small molecule activators and inhibitors.

TRPML1 currents have been recorded in endolysosomal patch-clamp experiments frequently in the past, for instance see: (Chen et al., 2017a; Chen et al., 2014; Plesch et al., 2018; Rühl et al., 2021). While it is clearly possible to determine TRPML1 currents with this method, endolysosomal patch-clamp technique always uses single isolated vacuoles and is therefore not suitable to quantify channel expression.
In terms of ML1-SA1, it has been intensively characterized in a recent publication (Spix et al.), showing selectivity of TRPML1 over TRPML2 or TRPML3 (see Figures 7E-H in Spix et al.).
We agree with the reviewer, that differences in sensitivity towards ML1-SA1 in different cell lines are more complex and cannot just be explained with different expression levels. If a clear statement should be drawn on the underlying mechanisms of differential sensitivity, then many further experiments would be necessary. Yet, this is not the focus of our study. We focused on the role of TRPML1 in mitochondrial regulation and therefore just picked a model in which we see sufficient ML1-SA1 sensitivity. We included an explanation in the manuscript, to clarify this.

4. Both ML1-SA1-induced TRMPL1 channel activation and depletion of TRPML1 expression resulted in similar effects on mitochondrial structure (Fig.3C-G) and mitochondrial membrane potential (Fig.4A), which are striking but how they are reconciled. In addition, the authors need to examine whether TRPML1-KO prevented ML1-SA1-induced effects on mitochondrial membrane potential (Fig.4A) and ATP production (Fig.4H).

We agree with the reviewer, that it would be interesting to evaluate the on-target effects of ML1-SA1 in more detail. Therefore, we included several experiments on TRPML1 KO cells within the revised manuscript, as suggested. The results are described and highlighted at the respective site in the revised manuscript.
In addition to the reviewer's suggestion, we analyzed the dose response of ML1-SA1 in TRPML1 KO cells. These results show, that the IC50 is significantly higher in KO cells (see Fig 2A new and Table 1 new).

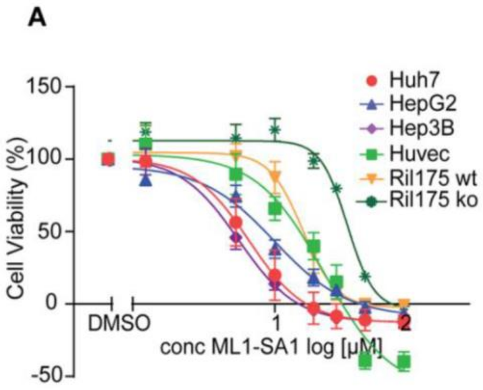

Figure 2 (revised graphs)

Cell line	IC50 [µM]	p-value[1]
Ril-175 wt	16.44	<0.0001
Ril-175 TRPML1KO	36.45	<0.0001
HUH-7	6.31	<0.0001
HepG2	9.97	<0.0001
Hep3B	5.24	<0.0001
HUVEC	24.09	<0.0001

Table 1 (revised)

This clearly depicts an on-target activity. Please note, that it is hardly ever possible to completely abolish effects on cell viability upon knock-out of the respective target. The reason for this lies in off-target effects, which all drugs show to a certain degree and the fact that effective drug concentration at these off-targets is higher, when the main target is absent.

© 2022. Published by The Company of Biologists under the terms of the Creative Commons Attribution License (https://creativecommons.org/licenses/by/4.0/).

We measured the mitochondrial membrane potential using JC1 staining in TRPML1 KO cells treated with ML1-SA1 in addition to the existing data. While the disruption of the membrane slightly increases upon drug treatment also in the KO cells, which can be seen as general stress response, it is not significantly altered. Yet, we still clearly find a significant mitochondrial membrane disruption in cells treated with 50µM ML1-SA1 and in KO cells compared to WT cells (see Fig 4A new). Similarly, we also measured ATP level in in TRPML1 KO cells treated with ML1-SA1 in addition to the existing data. Also in this regard, ATP levels in KO cells are not significantly changed upon ML1-SA1 treatment (see Fig 4H new).

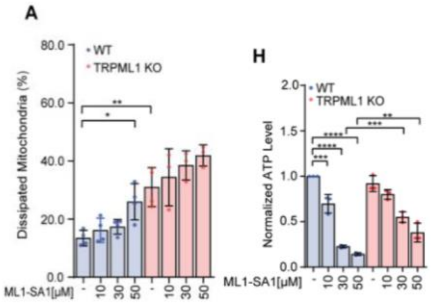

Figure 4 (revised graphs)

Figure legends have been adapted respectively.
These data show, that ML1-SA1 effects on mitochondria and ATP level are due to an on-target action on TPML1.

5. Application of ML1-SA1 or other TRPML1 activators elevated intracellular calcium and mitochondrial calcium (Fig.5A-E). It is imperative to know whether TRPML1-KO abolished such calcium responses. Similarly, experiments are required to examine whether TRPML1-KO prevented ML1-SA1-induced mitochondrial ROS production (Fig.5F), and mitochondrial stress indicated by increased OPA1cleavage and reduced elF2α phosphorylation (Fig.5G-I).

As with the previous comment, we agree with this one. Therefore, we have substantially revised this figure and included additional data on the effect of ML1-SA1 in TRPML1 KO cells.

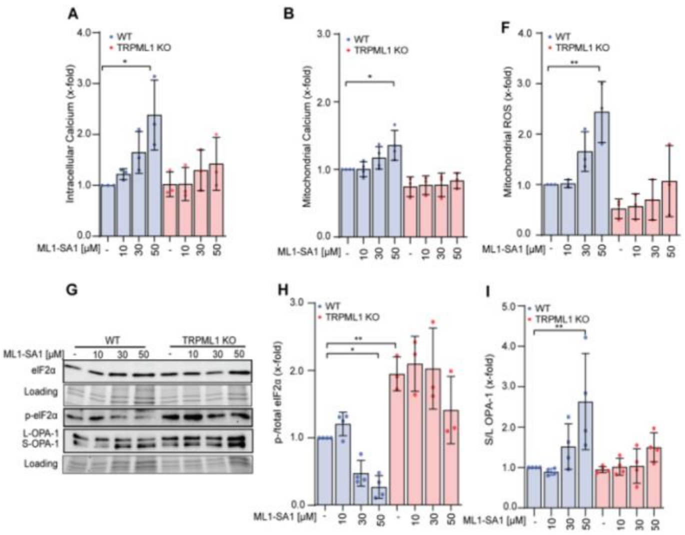

Figure 5 (revised graphs)

Measurement of intracellular calcium levels revealed that ML1-SA1 showed no significant effect in TPML1 KO cells (see Fig 5A new). The same holds true for mitochondrial calcium levels (see Fig 5B new) as well as mitochondrial ROS levels (see Fig 5F new) and eIF2a and OPA-1 protein levels (see Fig 5G-I new). Taken together, these findings further emphasize, that the observed effects are ML1-SA1 on- target effects, due to TRPML1 channel activation. Figure legends have been adapted respectively.

© 2022. Published by The Company of Biologists under the terms of the Creative Commons Attribution License (https://creativecommons.org/licenses/by/4.0/).

6. The results summarized in Fig.6 clearly indicate that genetic depletion of TRPML1 expression introduced considerable alterations in both lysosomal and mitochondrial functions. Further experiments are required, for example, using TRPML1 inhibitors, to clarify how these effects due to TRPML1-KO is dependent or independent of the TRPML1 channel activity.

We thank you for your suggestion and revised the data accordingly. As you can see from our novel supplementary figure S5 B-C, inhibition of TRPML1 function with EDME leads to reduction in proliferation as well as KO.

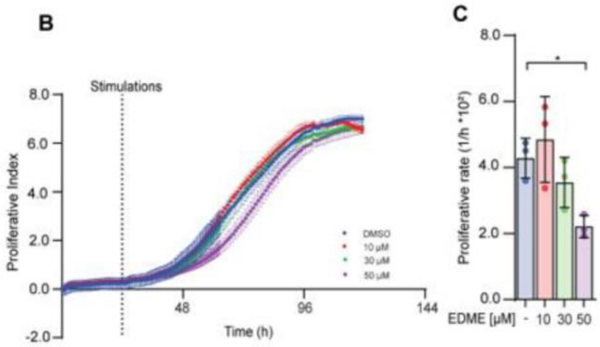

Figure S5 (revised graph)

Additionally, we've added data on mitochondrial as well as lysosomal structure and autophagy for pharmacological inhibition (see Figure 3 and 6 new). Staining with MitotrackerRed^{TM} revealed a similar phenotype as channel KO (see Figure 3C-D new).

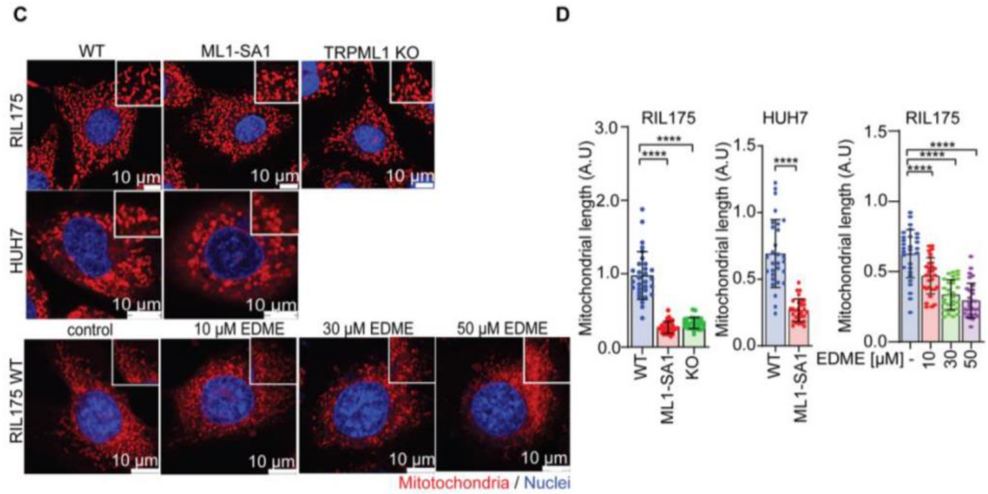

Figure 3 (revised graphs)

Accordingly, Lysotracker staining also led to the same phenotype as channel KO (see Figure 6E new). Inhibition of the channel with EDME led to an inhibition of autophagic flux and p62 degradation, as does TRPML1 KO. Further, treating KO cells with EDME did not have an additional effect on autophagy, indicating an on-target effect (see Figure 6G new).

© 2022. Published by The Company of Biologists under the terms of the Creative Commons Attribution License (https://creativecommons.org/licenses/by/4.0/).

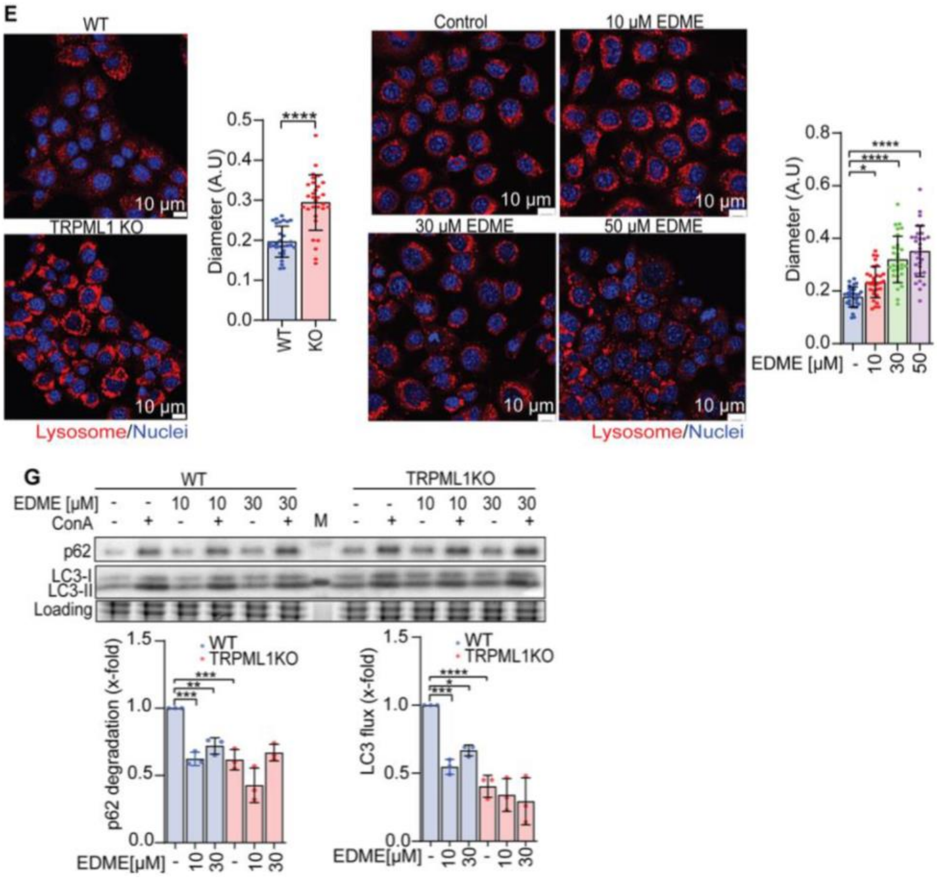

Figure 6 (revised graphs)

7. The mechanisms summarized in Fig.7 are not fully supported by the evidence from this study. For example, no evidence supports mitochondrial calcium is from calcium released from lysosome; mitochondrial calcium triggers opening of the mitochondrial transition pore; loss of mitochondrial membrane potential further increases mitochondrial calcium (left panel); and how TRPML1-KO causes lysosomal dysfunction (right panel).

We thank you for raising this concern. In the new, adapted figure, we have included several aspects from our study, as well as from literature (see Fig 7 new). Figure legends have been adapted accordingly. We hope that it now more clearly reflects the potential mechanisms that lead to mitochondrial dysfunction upon TRPML1 activity modulation.

© 2022. Published by The Company of Biologists under the terms of the Creative Commons Attribution License (https://creativecommons.org/licenses/by/4.0/).

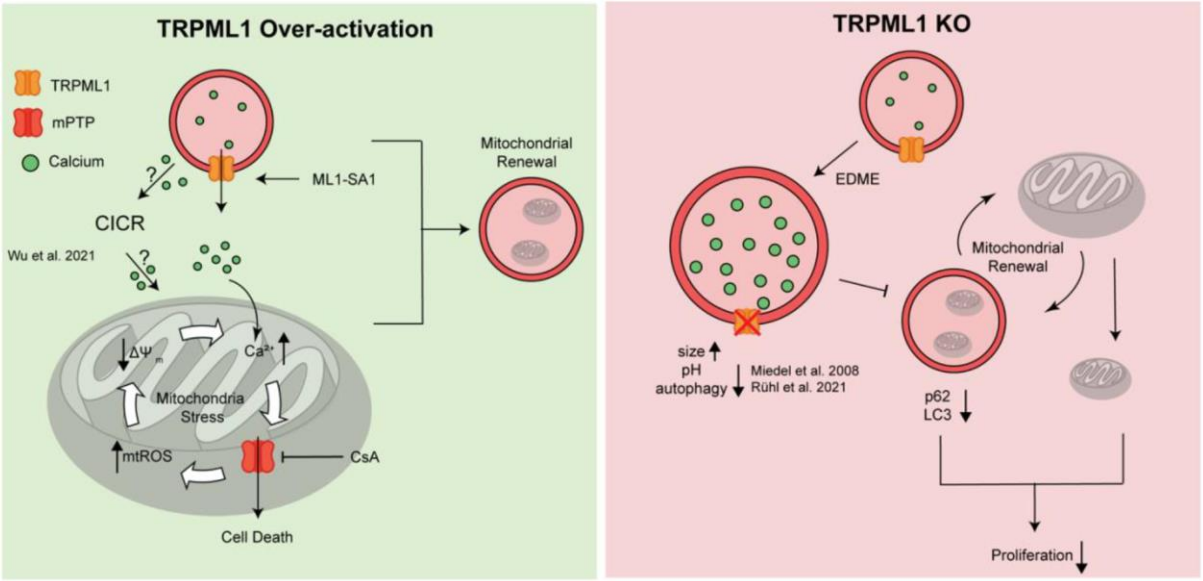

Figure 7. Model of TRPML1 mitochondrial balancer function in HCC. (Left panel) In TRPML1 wildtype cells calcium is stored in the endolysosomal system can be released by the channel. Induction of TRPML1 over- activation by ML1-SA1 leads to Ca2+ release into the cytosol. This calcium can either be and taken up by mitochondria directly, or induce calcium induced calcium release from other organelles, as for instance the endoplasmatic reticulum. Mitochondrial calcium uptake causes mitochondrial membrane depolarization and generation of ROS. This leads to an opening of the mitochondrial transition pore (mPTP) and subsequent cell death, which can be prevented by CsA. (right panel) Upon TRPML1 knockout, lysosomal function is impaired leading to a lysosomal swelling and impaired function. For instance, size as well as pH are altered. Subsequently, mitophagy and therefore mitochondrial renewal is disturbed. In sum, the impairment in mitochondrial and lysosomal function leads to a decreased cellular proliferation rate. TRPML1: transient receptor potential cation channel, mucolipin subfamily, mPTP: mitochondrial permeability transition pore, ML1-SA1: isoform-selective TRPML1 activator, mtROS: mitochondrial reactive oxygen species, CsA: cyclosporin A, ΔΨm: mitochondrial membrane potential

8. The rationale of the experiments is not compelling and the way of presenting the data is poor in logic. It would be more logical and much easier to follow if the results are presented for example in an order: TRPML1 expression and function; effects of TRPML1 activation on lysosomal and mitochondrial structure and function, and cell viability and proliferation; effects of TRPML1-KO or TRPML1 inhibitors on lysosomal and mitochondrial structure and function, and cell viability and proliferation; and, finally, TRPML1-KO or TRPML-1 inhibitors on TRML1 activator-induced effects on lysosomal and mitochondrial structure and function, and cell viability and proliferation

We thank the reviewer for this suggestion; however, we will maintain the structure of the manuscript. The alternative structure suggested would be starting with TPML1 expression and function. Yet, this is not in the focus of our study. There have been excellent manuscripts published on TRPML1 function, expression and localization and there are many reviews available on that topic see e.g. (Santoni et al., 2020a; Santoni et al., 2020b; Wang et al., 2014; Zeevi et al., 2007). Our aim was to develop a system in which we can study the biological role of TRPML1 activation or loss-of-function, which set the basis for our approach. Further, we based our hypothesis, that TRPML1 is important for mitochondrial function on an unbiased proteomic screening approach, rather than serendipity or individual findings in literature. We therefore think the experiments for system development and proteome should led the results part.

9. The Discussion session is a bit lengthy and speculative. More efforts are required to provide a clear interpretation of the results, and the significance of the finding from this study

We thank the reviewer for the remark and somewhat shortened the discussion to clearify interpretation. All changes have been highlighted in the text.

© 2022. Published by The Company of Biologists under the terms of the Creative Commons Attribution License (https://creativecommons.org/licenses/by/4.0/).

Minor points:

1. Describe the methods used in the order they appear in the Results session.

We thank the reviewer for the suggestion and restructured the methods as suggested, rather than alphabetically.

2. Provide the details of how to determine early and late apoptosis and necrosis in Fig.2C.

A description of determination of cell death stages has been included and marked in the main text of the manuscript. We included the following description:
"We next analyzed cell death induction by TRPML1 activation and KO (Fig. 2C) using Annexin V-FITC/PI staining and analysis by flow cytometry. Annexin V binds to phosphatidylserine residues which are flipped to the outer surface of the membrane early in apoptosis. Co-staining with PI, which labels cellular DNA allows differentiation between early apoptosis (Annexin V positive/PI negative), late apoptosis (Annexin V positive/PI positive) and necrosis (Annexin V negative/PI positive)."

3. The results in Fig.3A-B are limited in significance and better placed with related supplemental data.

We would like to clarify the significance of the findings in Fig. 3A-B. The proteome was performed in two ways, first wild-type DMSO treated vs. wild-type ML1-SA1 treated cells and second wild-type vs. TRPML1 knock-out cells (Fig. S2). The proteome analysis revealed significantly upregulated (red) or downregulated (blue) proteins in the volcano plots (Fig. S2 E-F). To assess the biological significance of the alterations, we performed a gene-set enrichment analysis of these proteins. This GSE analysis revealed that many proteins related to mitochondrial functional clusters are deregulated in ML1-SA1 treated cells, as well as in TRPML1 KO cells (Fig. 3A-B). We feel that showing the clusters of altered proteins underlines the rationale to check for the role of TRPML1 in mitochondrial function. We therefore prefer keeping the figure 3A and B in the main manuscript.

4. The writing needs substantial improvement in language expression and data presentation, for example, comparison of the data with to the literature should be in the Discussion rather session than the Results session!

We thank the reviewer for the time and effort invested in the review of our manuscript, as well as the helpful suggestions provided. We have carefully revised the manuscript and indicated any adaptation within the text in the revised version. We have also carefully checked the results part for literature references. Yet, we would like to point out, that references made to literature in this part are not meant to discuss, but rather to understand the rationale of the workflow or methodology.
References in 2.1. refer to the drugs used and methods, described previously. These have not been developed within this study.
References in 2.5 refer to knowledge from previous studies. We included these in the results part as to give evidence on the statement rather than speculate. The provided literature helps the reader to understand, why we analyzed OPA-1 and eIF2a phosphorylation during our investigation.
The reference in 2.6 refers to the mechanism of ROS detoxification by GSH, which has been studied and published elsewhere. This reference also helps to understand the rationale as to why we analyzed GSH and GSSG levels during our study.
We therefore feel, that including these few studies in the results part improve understandability of the study design. Further, in accordance with good scientific practice, we think it is important to state that these methods, tools, and findings where not conducted by ourselves.

Reviewer 2

Advance Summary and Potential Significance to Field:

Summary of study

Liver cancers, including hepatocellular carcinoma (HCC), are the second most lethal cancers worldwide and novel therapeutic strategies are still highly needed. Recently, the endolysosomal cation channel TRPML1 has gained focus in cancer research representing an interesting novel target The study utilized the recently developed isoform-selective TRPML1 activator ML1-SA1 and the

© 2022. Published by The Company of Biologists under the terms of the Creative Commons Attribution License (https://creativecommons.org/licenses/by/4.0/).

CRISPR/Cas9 system to generate tools for over-activation and loss-of-function studies on TRPML1 in HCC. The findings indicate that even though mitochondria are structurally affected by both channel activation and loss of function in a similar fashion, the functional consequences differ in both quantity and quality. The study suggests that activating TRPML1 by ML1-SA1 leads to morphological and functional changes in the mitochondria and subsequent dysfunction, caused by calcium overloading, which induces mitochondrial stress and mPTP opening. These results suggest that disturbance of TRPML1 might disturb the renewal of mitochondria and leads to decrease of cancer cell proliferation. In summary, the findings of the study suggest that targeting TRPML1 might be a promising approach to target HCC by manipulating mitochondrial function

My comments:
The study has produced some original and interesting findings of relevance to out understanding of cell biology and cancer therapy. The study is well executed and the report of the findings is well written and clear. The discussion makes some interesting points about the significance of the findings.

Reviewer 2 Comments for the Author:
I am happy with the manuscript and the findings of the study. The methodology is clear and the study well executed. The manuscript is well written.

We thank the reviewer for their time and effort invested in reviewing the manuscript and for the positive comments. Due to concerns raised by reviewer 1, we included additional aspects and literature in the discussion and hope that you find it still interesting or even improved.

Literature

Chen, C.-C., Butz, E. S., Chao, Y.-K., Grishchuk, Y., Becker, L., Heller, S., Slaugenhaupt, S. A., Biel, M., Wahl-Schott, C. and Grimm, C. (2017a). Small Molecules for Early Endosome- Specific Patch Clamping. *Cell Chemical Biology* **24**, 907-916.e4.

Chen, C. C., Cang, C., Fenske, S., Butz, E., Chao, Y. K., Biel, M., Ren, D., Wahl-Schott, C. and Grimm, C. **(2017b). Patch-clamp technique to characterize ion channels in enlarged individual endolysosomes.** ***Nat Protoc*** 12, **1639-1658.**

Chen, C. C., Keller, M., Hess, M., Schiffmann, R., Urban, N., Wolfgardt, A., Schaefer, M., Bracher, F., Biel, M., Wahl-Schott, C. et al. (2014). A small molecule restores function to TRPML1 mutant isoforms responsible for mucolipidosis type IV. *Nat Commun* **5**, 4681.

Plesch, E., Chen, C. C., Butz, E., Scotto Rosato, A., Krogsaeter, E. K., Yinan, H., Bartel, K., Keller, M., Robaa, D., Teupser, D. et al. (2018). Selective agonist of TRPML2 reveals direct role in chemokine release from innate immune cells. *Elife* **7**.

Rühl, P., Rosato, A. S., Urban, N., Gerndt, S., Tang, R., Abrahamian, C., Leser, C., Sheng, J., Jha, A., Vollmer, G. et al. (2021). Estradiol analogs attenuate autophagy, cell migration and invasion by direct and selective inhibition of TRPML1, independent of estrogen receptors. *Scientific Reports* **11**, 8313.

Santoni, G., Maggi, F., Amantini, C., Marinelli, O., Nabissi, M. and Morelli, M. B. (2020a). Pathophysiological Role of Transient Receptor Potential Mucolipin Channel 1 in Calcium-Mediated Stress-Induced Neurodegenerative Diseases. *Frontiers in Physiology* **11**.

Santoni, G., Santoni, M., Maggi, F., Marinelli, O. and Morelli, M. B. (2020b). Emerging Role of Mucolipins TRPML Channels in Cancer. *Frontiers in Oncology* **10**, 659-659.

Venkatachalam, K., Wong, C. O. and Zhu, M. X. (2015). The role of TRPMLs in endolysosomal trafficking and function. *Cell Calcium* **58**, 48-56.

Wang, W., Zhang, X., Gao, Q. and Xu, H. (2014). TRPML1: an ion channel in the lysosome. *Handb Exp Pharmacol* **222**, 631-45.

Zeevi, D. A., Frumkin, A. and Bach, G. (2007). TRPML and lysosomal function. *Biochimica et Biophysica Acta (BBA) - Molecular Basis of Disease* **1772**, 851-858.

© 2022. Published by The Company of Biologists under the terms of the Creative Commons Attribution License (https://creativecommons.org/licenses/by/4.0/).

Second decision letter

MS ID#: JOCES/2021/259455

MS TITLE: Lysosomal TRPML1 regulates mitochondrial function in hepatocellular carcinoma cells

AUTHORS: Wei-Xiong Siow, Yashar Kabiri, Rachel Tang, Yu-Kai Chao, Eva Plesch, Carola Eberhagen, Florian Flenkenthaler, Thomas Froehlich, Franz Bracher, Christian Grimm, Martin Biel, Hans Zischka, Angelika M Vollmar, and Karin Bartel
ARTICLE TYPE: Research Article

I am happy to tell you that your manuscript has been accepted for publication in Journal of Cell Science, pending standard ethics checks.

Reviewer 1

Advance summary and potential significance to field

The study investigated the role of the TRPML1 channel in the lysosome in the regulation of mitochondrial function in hepatocellular carcinoma (HCC) cells. The results showed pharmacological activation of TRPML1 and genetic depletion of its expression resulted in substantial structural and functional alterations in lysosome and particularly mitochondria, thereby impacting cell viability and proliferation.

Comments for the author

The authors have made efforts to address my comments, and the revision is overall satisfactory.

Reviewer 2

Advance summary and potential significance to field

I am happy with the changes that have been made.

Comments for the author

I am happy with the changes that have been made.

© 2022. Published by The Company of Biologists under the terms of the Creative Commons Attribution License (https://creativecommons.org/licenses/by/4.0/).
